# Snakes on the Balearic Islands: An Invasion Tale with Implications for Native Biodiversity Conservation

**DOI:** 10.1371/journal.pone.0121026

**Published:** 2015-04-08

**Authors:** Iolanda Silva-Rocha, Daniele Salvi, Neftalí Sillero, Jose A. Mateo, Miguel A. Carretero

**Affiliations:** 1 CIBIO Research Centre in Biodiversity and Genetic Resources, InBIO, Universidade do Porto, Vairão, Vila do Conde, Portugal; 2 CICGE, Centro de Investigação em Ciências Geo-Espaciais, Faculdade de Ciências da Universidade do Porto, Observatório Astronómico Prof. Manuel de Barros, Vila Nova de Gaia, Portugal; 3 Servei de Protecció d’Espècies, Govern de les Illes Balears, Palma de Mallorca, Spain; Consiglio Nazionale delle Ricerche (CNR), ITALY

## Abstract

Biological invasions are a major conservation threat for biodiversity worldwide. Islands are particularly vulnerable to invasive species, especially Mediterranean islands which have suffered human pressure since ancient times. In the Balearic archipelago, reptiles represent an outstanding case with more alien than native species. Moreover, in the last decade a new wave of alien snakes landed in the main islands of the archipelago, some of which were originally snake-free. The identification of the origin and colonization pathways of alien species, as well as the prediction of their expansion, is crucial to develop effective conservation strategies. In this study, we used molecular markers to assess the allochthonous status and the putative origin of the four introduced snake species (*Hemorrhois hippocrepis*, *Malpolon monspessulanus*, *Macroprotodon mauritanicus* and *Rhinechis scalaris*) as well as ecological niche models to infer their patterns of invasion and expansion based on current and future habitat suitability. For most species, DNA sequence data suggested the Iberian Peninsula as the potential origin of the allochthonous populations, although the shallow phylogeographic structure of these species prevented the identification of a restricted source-area. For all of them, the ecological niche models showed a current low habitat suitability in the Balearic, which is however predicted to increase significantly in the next few decades under climate change scenarios. Evidence from direct observations and spatial distribution of the first-occurrence records of alien snakes (but also lizards and worm lizards) suggest the nursery trade, and in particular olive tree importation from Iberian Peninsula, as the main pathway of introduction of alien reptiles in the Balearic islands. This trend has been reported also for recent invasions in NE Spain, thus showing that olive trees transplantation may be an effective vector for bioinvasion across the Mediterranean. The combination of molecular and ecological tools used in this study reveals a promising approach for the understanding of the complex invasion process, hence guiding conservation management actions.

## Introduction

Biological invasions are a major cause for current species extinctions, together with the destruction and fragmentation of habitats and climate change [1,2]. The rates of these processes, and in particular of biological invasion, have strongly increased during the last century due to human growth and globalization causing a parallel increase in the rate of biodiversity loss [3–5]. Thus, understanding the processes of introduction and expansion of invasive species has become a conservation priority [5].

Islands ecosystems are especially vulnerable to biological invasions since many insular native species have been evolving long time in isolation often losing their ability for competing with other species, eluding predators or defending themselves from parasites, which may arrive from nearby continents [6–9]. In particular, islands within the Mediterranean basin are distinctive due to the ancient interactions between biota and human activities which caused severe changes on the biodiversity present over time. Thus, many Mediterranean islands now share evolutionary lineages with the surrounding continents or other islands. The process is so intensive and widespread that even the distinction between allochthonous and autochthonous species may be problematic [10]. This is particularly true for reptiles, one of the groups most widely introduced in Mediterranean islands, but at the same most threatened by introductions [9,11].

**Fig 1:**
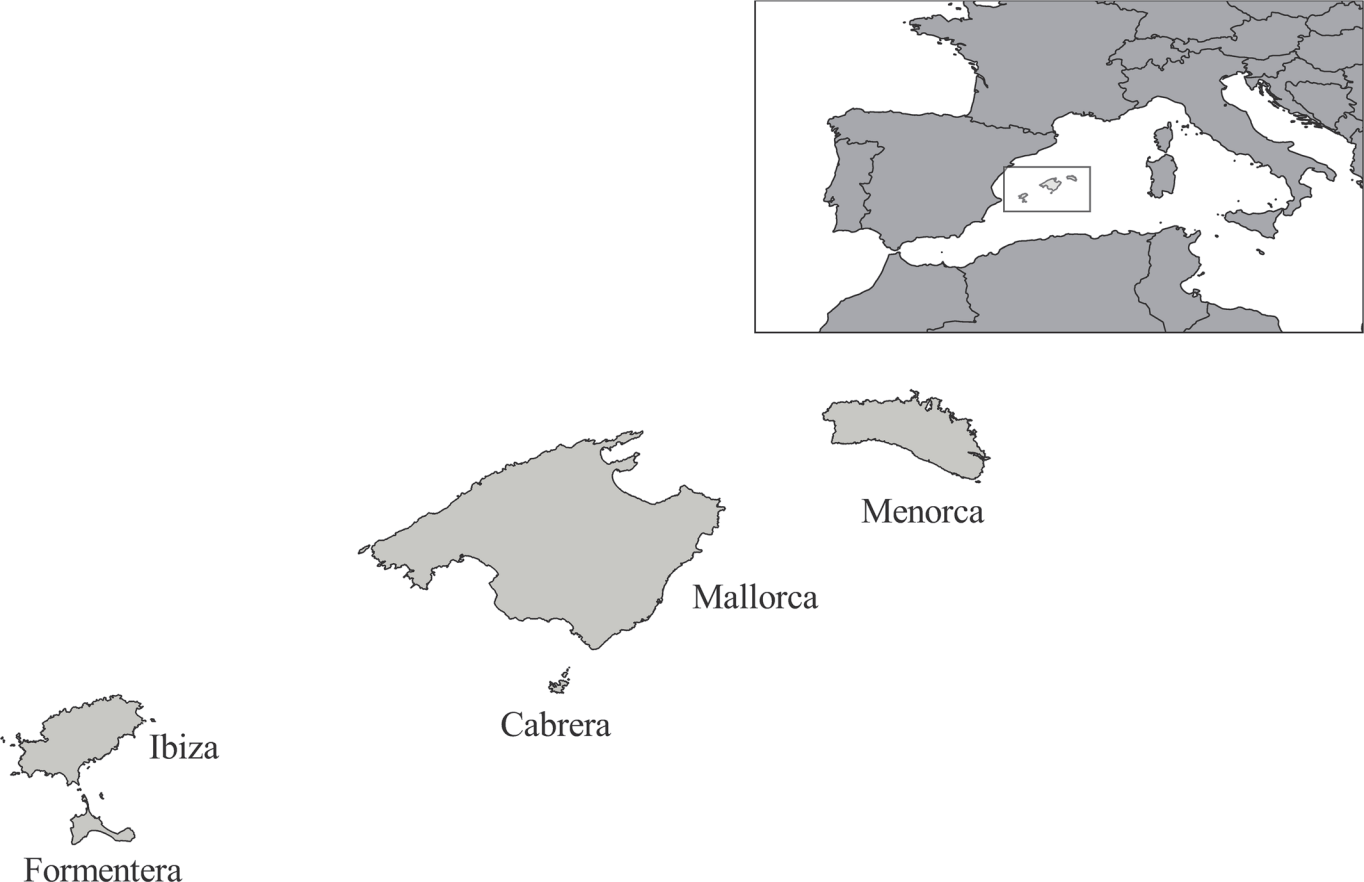
Map of the Balearic archipelago. The inset (top right) shows the geographic position of the Balearic archipelago in the Western Mediterranean.

In this respect, reptiles in the Balearic Islands (Mallorca, Menorca, Ibiza, and Formentera; see Fig. 1) represent the most paradigmatic case within the Mediterranean region and perhaps one of the outstanding cases in the world [12], with by far more alien (19) than native (2) species. Isolated from the continent since the Messinian (5.33 my BP) [13], this archipelago currently harbours only two endemic reptiles, the Lilford’s and Ibiza wall lizards (*Podarcis lilfordi* and *P*. *ptyusensis*, respectively) [12]. By contrast, a striking number of alien species have been reported for the main islands, namely, 16 in Mallorca, two in Cabrera (south of Mallorca), 11 in Menorca, six in Ibiza, and five in Formentera.

In this archipelago, the first reptile introductions date back to the Neolithic [12]: thus, that part of the alien species contingent of the Balearics was already naturalized and widespread in one or more islands since historical times (for a review see [12, 14]). The historical introduction of the snakes *Macroprotodon mauritanicus*, together with some introduced mammals, have been considered responsible for the extinction of native lizards *P*. *lilfordi* in Mallorca and Menorca [12, 15–17] and of the native Menorcan amphibians *Alytes muletensis* and an undescribed Discoglossid [18]. On the other hand, many other species such as the Red-eared Slider Turtle *Trachemys scripta*, the ocellated lizard *Timon lepidus*, and surprisingly four snakes, have been recorded only recently, i.e. in the last decade, in one or more Balearic islands, likely as a result of the intensification of human activity such as tourism and pet trade [12]. Among these ‘new invaders’, those that certainly deserve particular conservation attention are the introduced snakes. Indeed, while several Balearic islands such as Ibiza and Formentera has been until recently free of snakes, since 2003 the alien snakes *Hemorrhois hippocrepis*, *Malpolon monspessulanus*, and *Rhinechis scalaris* have been recorded in Ibiza, and the latter also in Mallorca and, very recently, in Formentera [19–21].

Alien snakes represent one of the main threat to the native Balearic biota, since they are effective predators capable to transform the community composition and ecosystem properties [5]. Most of the introduced species are partially saurophagous and potentially very harmful for the populations of the native endemic lizard *Podarcis pityusensis* from Ibiza and Formentera islands, which remained snake-free until recently. Moreover, some of these snakes are able to adapt their diet based on food availability on islands. For instance, Balearic populations of *Macroprotodon* seems to have shifted dietary habits from reptiles, consumed by the mainland snakes, to mammals, due to the extinction of the endemic lacertid lizards from Mallorca [22]. This reflects the ability of these snakes to adapt to island environments and to have a wider potential impact on native species than expected according to their realized niche on mainland. Additionally, snakes are able to use human manufactures and ornamental plants as a refuge [23,24] which favours both their introduction and the development of populations. Remarkably, the number of alien snake observations in the Balearics has dramatically increased during the last decade [19], suggesting a range and population size expansions and/or recruitment through new introductions. Thus, understanding the origin and pathways of introduction of these alien species as well as forecasting their future expansion is extremely urgent for setting conservation actions.

In this study, we analysed in a phylogeographic framework mitochondrial DNA sequence data of the snakes *Hemorrhois hippocrepis*, *Malpolon monspessulanus*, *Macroprotodon mauritanicus* and *Rhinechis scalaris* reported for the Balearic Islands to assess their allochthonous status and putative origin and to infer pathways of introduction. Moreover, in an attempt to assess their spatial patterns of invasion and expansion in the Balearics, we estimated for each species the habitat suitability and the occurrence of environmental limiting factor at present and under future climatic scenarios using ecological niche models.

This integrated approach, joining molecular data and ecological models, represents a promising tool for the understanding of the complex invasion process, hence, guiding conservation management actions.

## Materials and Methods

### Study Area

The Balearic Islands have a total area of 5040 km^2^ and 1428 km of coastline, laying 80 to 300 km east of the Iberian Peninsula. This archipelago is composed by two islands groups: the Gymnesic (eastern) and the Pityusic (western) (see Fig. 1). The Gymnesic group is constituted by the main islands of Mallorca and Menorca, the small island of Cabrera (south of Mallorca) and about 30 surrounding islets. The Pityusic group includes Ibiza and Formentera islands and about 60 surrounding islets. The Balearic Islands are covered by typical Mediterranean vegetation with more diversity of ecosystems and landscapes in Mallorca due to its wider altitudinal range (highest altitude 1445 m). Introduced reptiles are currently present on the main islands (Mallorca, Menorca, Ibiza and Formentera) and not in the small islets.

### Ethical statement

Tissue samples for genetic analyses were collected from road kills animals by us or by technicians of the Balearic Government, thus no live animals have been damaged or sacrificed for this study. Samples have been collected according to the permits issued by the Balearic Government (CAP 55/2011), which approved all animal procedures used in this study.

### Molecular data gathering

In order to assess the allochthonous status and putative origin of the snakes *H*. *hippocrepis*, *Malpolon monspessulanus*, *Macroprotodon mauritanicus*, and *R*. *scalaris*, currently occurring in the Balearics, we *compared DNA sequences generated for Balearic specimens with those published for conspecific individuals from the native range in previous phylogeographic studies*: *H*. *hippocrepis* [25]; *Malpolon monspessulanus* [25]; *Macroprotodon mauritanicus* [26,27]; and *R*. *scalaris* [28]. *Note that* we followed the taxonomy proposed by Carranza et al. [26] and the Reptile Database (http://reptile-database.reptarium.cz/), that list four *Macroprotodon* species: *M*. *cuccullatus*, *M*. *brevis*, *M*. *mauritanicus* and *M*. *abubakeri*.

*Tissue* samples for genetic analyses were collected in all the main Balearic Islands from live and road-killed animals either directly by the authors during a field session in 2011 or thought the collaboration with local environmental authorities (see acknowledgments and ethical statement paragraph). Samples were stored in pure ethanol until total genomic DNA was extracted following the standard saline method [29].

We amplified fragments of the mitochondrial gene *cytb* from 34 Balearic specimens of all the four snake species. Additionally, for *H*. *hippocrepis* and *Macroprotodon* sp., a fragment of the ribosomal gene *12S* was also amplified (details on gene fragments size and primers used are indicated in Table 1). Amplifications were conducted by the Polymerase Chain Reaction method (PCR), using conditions reported in previously published phylogeographies of these species [25–28]. PCR products were purified and sequenced by an external service (company Macrogen Korea).

**Table 1:** List of primers used for amplification and sequencing of selected gene fragments for each species, with indication of fragment size.

Species	Gene	Primer	Size (bp)
*Hemorrhois hippocrepis*	*cytb*	Cytb1 and Cytb2 (Palumbi 1996)	241
	*12S*	12Sa and 12Sb (Kocher *et al*. 1989)	219
*Malpolon monspessulanus*	*cytb*	Cytb1 and Cytb2 (Palumbi 1996)	254
*Macroprotodon sp*.	*cytb*	GluDG and Cytb2 (Palumbi 1991)	250
	*12S*	12Sa and 12Sb (Kocher *et al*. 1989)	311
*Rhinechis scalaris*	*cytb*	GluDG and Cytb2 (Palumbi 1991)	353

Sequence data generated from the native range of each species, as well as the outgroup taxa, were mainly obtained from GenBank from previous phylogeographic studies (see above). Additionally, for *R*. *scalaris* we generated *cytb* sequences for 10 specimens from the native range in order to extend the preliminary phylogeographic assessment by Nulchis et al. [28] which was based on only 12 samples. A complete list of the sequences used in this study along with locality and GenBank Accession numbers is provided in S1 Table.

### Molecular data analysis

The sequences generated in this study were aligned with those gathered from GenBank using CLUSTALW [30]. For those species for which *cytb and 12S* were sequenced, we generated also a concatenated *cytb+12S* alignment. In order to infer the genealogical relationships between the Balearic samples and those from the native range of each species, we analysed *cytb* and *cytb*+*12S* datasets using the Maximum Likelihood (ML) and the statistical parsimony network methods. The ML analyses, as well as the estimation of the best model of sequence evolution, were performed in MEGA5 with the heuristic search mode [31]. Node support was calculated over 1000 bootstrap replicates. Statistical parsimony networks were estimated using the software TCS 1.21 [32].

### Distribution and environmental data gathering

We collected occurrence data for each species and environmental data for their native range in order to assess habitat suitability using ecological niche models.

We compiled occurrence data of the global native distribution of each species from GBIF (Global Biodiversity Information Facility, www.gbif.org), and cleaned from obvious data errors. For the species with North African distribution (i.e. all species except *R*. *scalaris*), more occurrence points were needed to conduct a proper analysis, since the information contained on GBIF database was not enough. In these cases, we collected additional geographic records from literature review, CIBIO database, and National Atlases from Portugal, Spain and Morocco [33–35]. We collected a total of 2953 records for *H*. *hippocrepis*, 7164 for *Malpolon monspessulanus*, 796 for *Macroprotodon* sp., and 6152 for *R*. *scalaris*. For *Macroprotodon* sp., we considered the presence records from the whole genus from Morocco, Algeria and Tunisia since the distribution of the species as proposed by [36] included very few records while the ascription of many others would have remained doubtful. We are aware that modelling the genus as a whole could lead to results not adjusted to the habitat characteristics of the particular species. However, by performing a join analysis of the genus we intended to obtain the maximum expected habitat suitability, provided that there would be niche conservatism within the genus. Whatever other case (i.e. niche differences between the putative species, with more arid or mesic affinities) would result in more restricted habitat suitability and thus in a lower predicted expansion. Hence, the suitable habitat for the particular species will be always included in the suitable habitats for the whole genus. We consider that this conservative procedure is advisable when dealing with invasive species and incomplete biogeographic information.

We downloaded climatic predictors from WorldClim v. 1.4 online data (www.worldclim.org, [37]) at a resolution of 30 arc-seconds (~ 1 km^2^). We selected a total of seven bioclimatic variables (from a total of 19) with a Spearman's correlation lower than 0.7 and meaningful for the species to calculate the realised niche models (*sensu* [38]): Bio2—Mean Diurnal Range; Bio3—Isothermality; Bio4—Temperature Seasonality; Bio8—Mean Temperature of Wettest Quarter; Bio15—Precipitation Seasonality; Bio18—Precipitation of Warmest Quarter; and Bio19—Precipitation of Coldest Quarter. For future climate scenarios, we used three coupled atmosphere-ocean general circulation models (CCCMA, HadCM3 and CSIRO) with three socio-economic emission scenarios (A1b, A2a and B2a), for three future periods: 2020, 2050 and 2080. As we only want to evaluate the tendency over the years, an average model was made with all the scenarios.

### Ecological Niche Model analyses

We modelled the realised ecological niche of each native range species (hence excluding the invasive range) at present and then projected to the present and future climatic scenarios of the Balearic Islands. This is the most correct methodology to avoid relying on species distribution data for areas where a lack of equilibrium is expected due to recent introduction (i.e. the Balearic islands), since we considered that the species are in pseudo-equilibrium (i.e. species must occupy all suitable habitats available, [39]). To calculate the models we use the Maximum Entropy method implemented in the software Maxent 3.3 (www.cs.princeton.edu/~schapire/MAXENT). Maxent is a general-purpose machine learning method that uses presence-only occurrence data and is consistently competitive with the highest performing methods [40,41]. Maxent output represents the habitat suitability, ranging from 0 to 1. We run Maxent with autofeatures, excluding the ‘product feature’, selecting at random 70% of the presence records as training data and 30% as test data for each species. The final model and projections for each species were the average of 10 different replicative models. Standard deviation was also calculated to determine the uncertainty of the models.

To maximize the predictions, we built models with an increasing regularisation parameter (β) for each species. The regularisation multiplier allows constraining the model to over-parameterization, and hence increasing values of β is expected to achieve the model with less complexity [42]. For all the species, we started with β = 1 and increased β+2 until we reached consistent results (*H*. *hippocrepis*: β = 61; *Macroprotodon* sp.: β = 43; *Malpolon monspessulanus*: β = 13; *R*. *scalaris*: β = 21). To compare all the models with the different β, we calculate the AICc values with ENMTools 1.3 [42].

We tested the quality of the models by calculating the area under the curve (AUC) of the receiver operated characteristics (ROC) plots [43] in order to discriminate a species' model from a random model. The importance of each climatic variable for explaining the species’ distribution was determined by jack-knife resampling: (1) of the training and test gain; and (2) of AUC values. For this purpose, environmental variables were excluded in turn and a model created with the remaining variables; then a model was created using each individual variable. Finally, we obtained an average percentage contribution of each environmental factor to the models.

A MESS (Multivariate Environmental Similarity Surfaces) analysis was also performed in order to detect non-analogous environmental conditions. This analysis measures the similarity of any given point to a reference set of points, regarding the chosen predictor variables [44].

We reclassified the projected models into habitat suitability maps (*sensu* [38]) according to the maximum training sensitivity plus specificity logarithmic thresholds given by Maxent. Cells with values higher and lower than the threshold were considered respectively as suitable and unsuitable (i.e. the species was considered absent) for the presence of the species.

## Results

### Genetic analyses

We obtained sequences for a total of 34 Balearic specimens of the four snakes: 24 *H*. *hippocrepis* (19 from Mallorca and five from Ibiza); one *Malpolon monspessulanus* from Mallorca; five *Macroprotodon* sp. (four from Mallorca and one from Menorca); and four samples of *R*. *scalaris*. A total of 10 *cytb* sequences of *R*. *scalaris* from the native range were also generated. These sequences were analysed together with sequences from Nulchis et al. [28] downloaded from Genbank (see S1 Table for further details).

**Fig 2:**
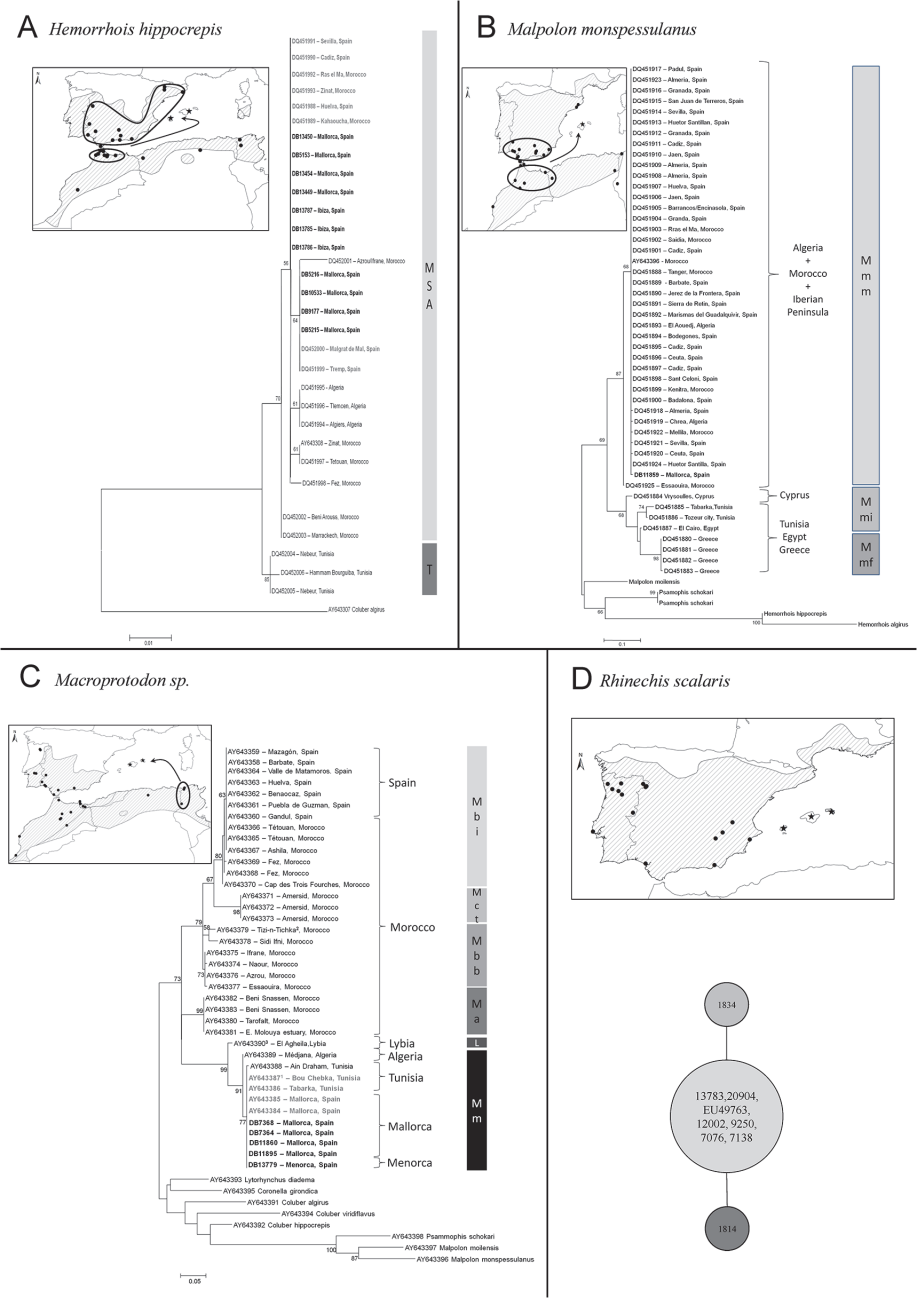
Results from genetic analysis and geographic origin of the introduced populations. Numbers on branches indicate ML bootstrap values (BP) over 1000 replicates (BP<50 are not reported). (2A): ML tree based on of the combined *12S*+*cytb* dataset depicting the relationships between haplotypes of the native range of *Hemorrhois hippocrepis* from Carranza et al. [23] and those from the introduced populations from Mallorca and Ibiza Islands. MSA: Morocco+Iberia+Algeria; T: Tunisia. (2B) ML tree based on of the *cytb* dataset depicting the relationships between haplotypes of native range of *Malpolon monspessulanus* from Carranza et al. [23] and those from the introduced populations from Mallorca Island. Mmm: *Malpolon m*. *monspessulanus*; Mmi: *Malpolon m*. *insignatus*; Mmf: *Malpolon m*. *fuscus*. (2C) ML tree based on of the *cytb* dataset depicting the relationships between haplotypes of native range of *Macroprotodon* sp. from Carranza et al. [24] and those from the introduced populations from Mallorca and Menorca Islands. Mbi: *Macroprotodon brevis ibericus*; Mct: *Macroprotodon cucullatus textilis*; Mbb: *Macroprotodon brevis brevis*; Ma: *Macroprotodon abubakeri*; L—Libyan clade; Mm: *Macroprotodon mauritanicus*. (2D) Statistical parsimony network depicting the genealogical relationships between *cytb* haplotypes from the native range and from Balearic individuals (Ibiza: 12002; Mallorca: 9250; Menorca: 7076 and 7138) of *R*. *scalaris*.

The genetic analysis of *H*. *hippocrepis* revealed two different haplotypes, both in *cytb* and *12S*+*cytb* datasets, among the Balearic samples, corresponding to those already found in the native range by Carranza et al. [25] (Fig. 2A). Snakes from Mallorca and Ibiza are related to those most commonly found in Spain and Morocco. Therefore, the genetic data do not allow to distinguish the exact source.

We identified a total of 14 haplotypes for native and Balearic samples of *Malpolon mospessulanus*, 13 of them corresponding to haplotypes previously found in samples from Carranza et al. [25] and one corresponding to the Mallorcan sample (Fig. 2B). The genetic analysis revealed two main clades: one including Moroccan and Iberian Peninsula samples belonging to *Malpolon m*. *monspessulanus*; other including Tunisian, Cyprian, Egyptian, and Greek samples corresponding to *Malpolon insignitus insignitus* and *Malpolon m*. *fuscus* (see [25]). The sample from the Balearics clustered together with *Malpolon m*. *monspessulanus* from Morocco and Iberia.

The genetic analyses of *Macroprotodon* sp. identified a single haplotype among the Balearic samples, identical to those previously found in two Tunisian samples and one Mallorcan sample by Carranza et al. [26] and ascribed to *Macroprotodon mauritanicus* clade (Fig. 2C). Finally, we found three *cytb* haplotypes in *R*. *scalaris*, one corresponding to the commonest haplotype widespread across the species’ range identified by Nulchis et al. [28] and two occurring in two localities of the native range analysed in this study. Due to the low genetic differentiation between the three haplotypes (one or two nucleotide differences), we show their genealogical relationships by a network, since the ML tree was less informative (not shown). The Balearic samples carried the most common haplotype widespread across the native species’ range (Fig. 2D).

### Ecological Niche Modelling

The AUC values were higher than 0.80 for all the species—*H*. *hippocrepis* 0.858 ± 0.004; *Macroprotodon* sp 0.902 ± 0.004; *Malpolon monspessulanus* 0.802 ± 0.001; and *R*. *scalaris* 0.814 ± 0.002—indicating a good performance of the models.

The contribution of each bioclimatic variable to species models is reported in Table 2. For *R*. *scalaris*, *H*. *hippocrepis* and *Macroprotodon* sp., the variable explaining most of the variation in the models was the precipitation of warmest quarter (bio18). Regarding *Malpolon monspessulanus*, the temperature seasonality seems to be the variable that more contribute to the models. The second most explanatory variable varied with the species: the precipitation seasonality (bio15) for *H*. *hippocrepris* and *Macroprotodon* sp., the precipitation of warmest quarter (bio18) for *Malpolon monspessulanus* and the temperature seasonality for *R*. *scalaris*. These patterns were concordant after jack-knife resampling (S1 Fig.).

**Table 2:** Contribution of each bioclimatic variable for each species’ model.

Variable	*Hemorrhois hippocrepis*		*Malpolon monspessulanus*		*Macroprotodon sp*.		*Rhinechis scalaris*	
	PC	PI	PC	PI	PC	PI	PC	PI
Bio2	2.5	6.7	9.6	15	12.6	12.6	18.4	21.2
Bio3	0	0	3.5	4.3	0	0.1	2.7	2
Bio4	6.9	2.7	*28*.*8*	*13*.*7*	2	0.5	*28*.*4*	*15*.*3*
Bio8	0	0	0.5	3.8	0.9	2.5	0.1	1.9
Bio15	22.8	0.6	8.3	6.7	*17*.*2*	*0*	12.3	5.3
Bio18	*39*.*3*	*71*.*5*	*29*.*8*	*36*.*3*	*57*.*2*	*81*	*37*.*6*	*44*.*5*
Bio19	*28*.*6*	*18*.*5*	19.6	20.2	15.7	3.4	0.5	9.8

The two main explanatory variables for each species are in italic. PC: Percentage contribution; PI: Permutation Importance. Bio2: Mean Diurnal Range; Bio3: Isothermality; Bio4: Temperature Seasonality; Bio8: Mean Temperature of Wettest Quarter; Bio15: Precipitation Seasonality; Bio18: Precipitation of Warmest Quarter; Bio19: Precipitation of Coldest Quarter.

Regarding the MESS analysis, we obtained negative values for all the present models, which means a high degree of dissimilarity between any given point and the distribution of reference points. However, the variables showing dissimilarity are also those that less contribute to the model (bio_3 and bio_15, S3 Fig.), with exception of *Macroprotodon sp*. for which bio_15 is the second most explanatory variable, however it contributes much less than bio_18. For the future models, we obtained positive values for all the species with none dissimilar variable (S3 Fig.).

### Suitability of Balearic Islands for invasive snakes

**Fig 3:**
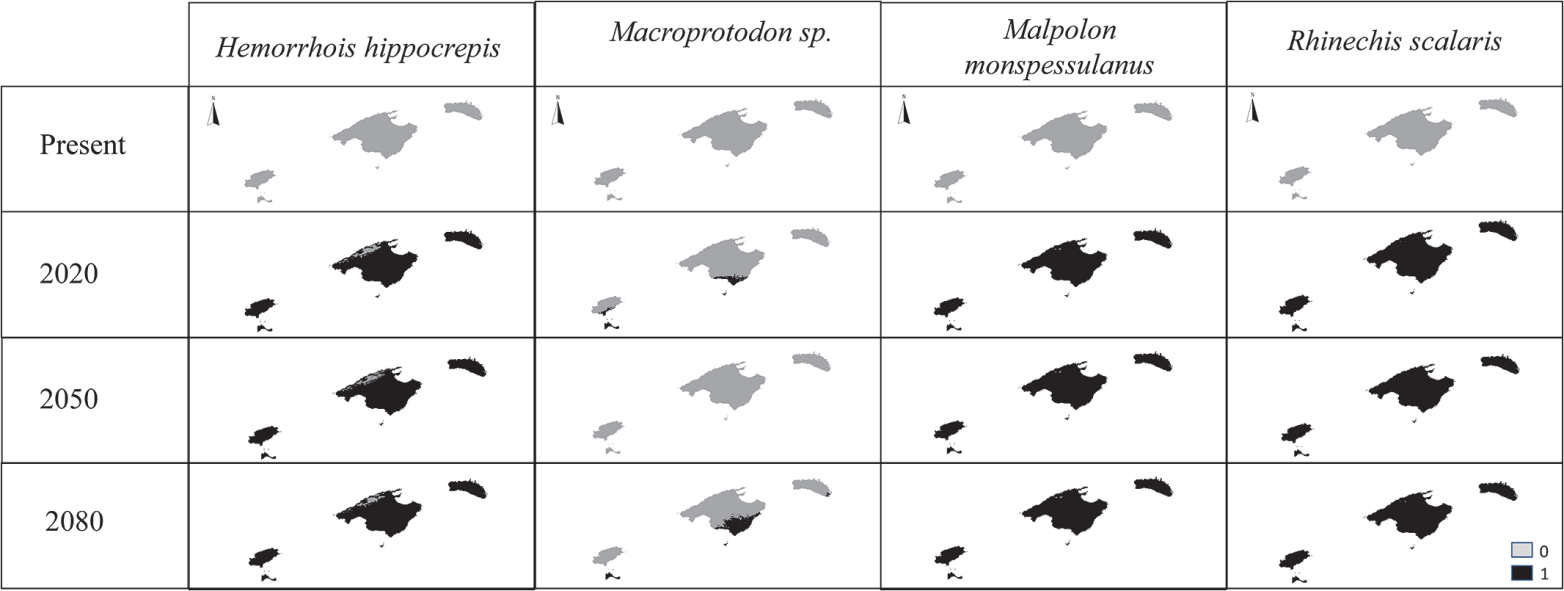
Habitat suitability models for the present and for the future (2020, 2050 and 2080) of all the study species.

In general, the models projected to the Balearic Islands showed a limited suitability in the present for the four snakes at the given occurrence threshold (see below; Fig. 3). Nevertheless, it is worth noting that known occurrence points from each species fall within unsuitable areas. Moreover, in the projections for the future, Balearic Islands become more suitable for the all the species, with exception of the smooth false snake (*Macroprotodon* sp.) which only might occupy Formentera, south of Mallorca and Cabrera (Fig. 3). Both *R*. *scalaris* and *Malpolon monspessulanus* attained the highest suitability probabilities and might occupy almost all the extension of all the islands. The horseshoe whip snake (*H*. *hippocrepis*) might be widespread on the island with exception of the Serra Tramuntana mountain range. It is important to remark that the habitat suitability index was relatively high in the future models (with exception of *Macroprotodon* sp.), being the maximum values 0.57 in 2020 for *H*. *hippocrepis*, 0.34 in 2080 for *Macroprotodon sp*., 0.60 in 2080 for *R*. *scalaris* and 0.59 in all the three years for *Malpolon monspessulanus*.

## Discussion

To protect Balearic endemic biota from contemporary biological invasions we have first to understand the current dynamic of this process and its possible future development. In particular, to plan effective measures aimed to prevent further introductions it is crucial to know, first, where are these aliens from, and, second, which is the pathway through which they arrive in the Balearic Islands. On the other hand, regarding those snakes that are already established in the archipelago, management measures rely on the knowledge on their current and future spatial patterns of distribution. In the following sections we discuss the results of this study following these three main points and, finally, addressing their conservation implications.

### Timing and origin of the snake introductions

The occurrence records and previous literature allow distinguishing between species historically present in the archipelago and those arrived only very recently (last decade). The first group (historical introductions) includes the populations of *Macroprotodon* sp. from Mallorca and Menorca, and *R*. *scalaris* from Menorca. Previous studies suggest introduction during Roman times for both the species, however it is important to stress that this is just a hypothesis [34,45–47]. The second group (recent introductions) includes *H*. *hippocrepis* and *Malpolon monspessulanus* from Ibiza and Mallorca and *R*. *scalaris* from Ibiza, Mallorca and Formentera. In fact, for *H*. *hippocrepis* and *Malpolon monspessulanus*, the first observations in Ibiza only date from 2003 [19]; for *R*. *scalaris*, observations date from 2003 in Ibiza, 2004 in Mallorca, and 2006 in Formentera [19–21].

Phylogeographic analyses of all the species sampled in the Balearic Islands confirm their allochthonous status, suggesting an origin from North African or Iberian populations. Genetic evidence supports a Tunisian origin for Mallorcan and Menorcan population of *Macroprotodon* sp. (historical introduction; Fig. 2C), which therefore were assigned to *Macroprotodon mauritanicus* following [36]. This result is in agreement with the morphological identification of 168 *Macroprotodon* individuals recorded in the Balearic Snakes Database of the Servei de Protecció d'Especies of Govern Balear (124 records from Mallorca, 44 from Menorca). Moreover, a Tunisian origin for Mallorcan *Macroprotodon* snakes was already proposed by Busack and McCoy [46], Wade [36], and Carranza et al. [26], on the basis of the morphological and genetic similarity between Mallorcan and Tunisian specimens. In the case of *H*. *hippocrepis* and *Malpolon monspessulanus* (recent introductions), genetic data do not unambiguously differentiate between North-western African and European origins (Fig. 2A and 2B, respectively), given the extreme genetic similarity between native populations from these two regions [25]. Indeed, for both species, a recent expansion during Late Pleistocene from North-eastern Africa to Iberia has been suggested to explain the slightly higher genetic diversity observed in the North African populations of these species and their overall similarity with Iberian populations [25]. However, after analysing the phylogenetic tree of *H*. *hippocrepis*, Mallorcan samples clustered in a sub-haploclade including haplotypes found only in the Iberian Peninsula. This suggests an Iberian origin for *H*. *hippocrepis* from Mallorca, yet a North African origin cannot be completely discarded. Besides the phylogenetic evidence, the finding of this species in Mallorca near ‘nurseries’ of olive trees [19] (J. A. Mateo pers. obs.) that arrived from southern Spain further supports an Iberian origin of Mallorcan populations (see the section ‘Pathways of introduction’ for further details).

Phylogenetic network analyses show that the haplotype of *R*. *scalaris* found in Menorca (historical introduction), Mallorca, and Ibiza (recent introductions) is identical to the commonest haplotype found across the species’ range, suggesting a recent arrival to the Balearic archipelago from the mainland (Fig. 2D). Yet it was not possible to point a restricted source-area given the lack of phylogeographic structure of this species [28], while the genetic identity between all the Balearic populations and the continent allows discarding an ancient (i.e. Pleistocenic) colonization of the archipelago. Therefore, summarizing an allocthonous status of Balearic snakes was confirmed for both historical and recent introductions, being the latter most likely originated from Iberia.

The high genetic homogeneity of Balearic individuals of *H*. *hippocrepis* (two similar haplotypes), *Macroprotodon mauritanicus* and *R*. *scalaris* (one single haplotype) would suggest that each species invaded the Balearic Islands from a single origin. However, the very shallow mitochondrial phylogeographies of these species hampered the identification of restricted source-areas and therefore of multiple-origin patterns. For this purpose, it will be necessary the use of more variable markers (e.g. microsatellites) across the native and the Balearic ranges of these species, possibly with additional Balearic samples.

### Pathways of introduction

The introduction pathway for all the species is clearly human-mediated. In the case of the older introductions, such as the Menorcan population of *R*. *scalaris*, and the Mallorcan and Menorcan populations of *Macroprotodon mauritanicus*, the anthropogenic origin is supported by the absence of fossil record and the low genetic differentiation between the islands and the mainland populations [12,19,24,47]. The main pathways by which these species arrived to the Balearic Islands in historical times can be either voluntary or accidental. Among the voluntary factors, ancient Greek and Roman religious purposes cannot be discarded, since snakes appear to have a wide range of religious associations (i.e. Asklepios/Aesculapius cult), namely as guardians of a place, to bring luck, and to reveal if a place was sacred or not [48,49]. Moreover, snakes were also used by ancient Greeks, Carthaginians and Romans to frighten enemies during assaults [50]. Therefore, a probable voluntary introduction is favoured, since the humans likely obtained direct benefits from the translocation of snakes in these islands. Among the accidental factors, one possible way of introduction may have been the involuntary transport via shipments during an intense maritime trade along commercial routes established since long times between Balearic Island and the mainland. Many species use the human manufactures as a refuge, making more probable its accidental translocation from the native range toward the introduced range.

While historical pathways of introduction are difficult to disentangle, the inference of recent introductions can be based on the large amount of information we collected joining bibliographical and Government entities data. Commercial shipments by sea is most likely the main pathway for the recent biological invasions observed in the Balearic Islands. Mounting evidence strongly indicates that several alien reptiles (not only snakes) arrive in the Balearic islands through the ‘nursery trade’, which refers to the trade in live plants for ornamental purposes [11,19]. Most of the records of *H*. *hippocrepis*, *M*. *monspessulanus*, and *R*. *scalaris*, and significantly their first appearance in the Balearic Islands, have been recorded by environmental authorities inside trunks or root balls of olive trees deposited in the nursery centres of Capdepera (Mallorca) and Sant Llorenç de Baláfia (Ibiza) [19] (Balearic Snakes Database of the Servei de Protecció d'Especies del Goberno Balear, BSD-SPEGB). In the former centre, an adult individual of *H*. *hippocrepis* was found by one of us (J. A. Mateo) inside the trunk of an old olive tree just arrived from Córdoba province (Andalusia, southern Spain). Moreover, nursery centres are the places where several alien reptiles have been recorded for the first time in the Balearic during the last decade, namely the amphisbaenian *Blanus cinereus* (BSD-SPEGB; J. A. Mateo, personal observation), the blind snake *Indotyphlops braminus* (BSD-SPEGB) and the ocellated lizard *Timon lepidus* (Mallorca Reptile Database of COFIB, MRD-COFIB).

The nursery trade has increased in importance since the 1970’s, and accordingly this trade has increased its importance as vector of biological invasions [11]. This is particularly true for olive trees translocation across the Mediterranean [14,19,51–53], (this study). While formerly olive trees were moved across this region due to olive oil production, nowadays old trees are being transplanted also as decorative plants for gardens. Paradoxically, this trend is somehow in line with the European Agricultural Policies which encourage the removal of old, less productive, trees [54]. It is worth noting that only continental trees from Spain (Córdoba, Sevilla, Jaen, and Valencia provinces) and in small amounts from Southern Portugal (E. Ayllón com. pers.) are used to fulfil the gardening demands of the Balearics. No olive tree importations from North Africa are known. For instance, one of the nurseries in Mallorca imported 4000 olive tree specimens of different sizes since 1992 [54], which reflects the big market associated with these plants.

Several factors make the olive trade a powerful vector for biological invasions across the Mediterranean. First, olive trees are normally uprooted and transported during the winter because these practices require the trees to be in the latency phase. Second, the uprooting procedure implies the removal of a very large root ball—about 2 m^3^—around the olive tree. Third, both the plant nurseries that import these olive trees and the gardens where they are implanted are generally located in areas with Mediterranean conditions, i.e. with a similar climate to the place of origin. These three factors make the *time*, the *mode*, and the *destination* of olive tree translocations, particularly suitable for the transport, survival, and naturalization in the new environment of the entire ark of organisms that use the olive tree roots and cavities as a winter refuge (see also [11]). Indeed, old olive trees are hollow and able to carry several species of reptiles including snakes, lizards, and geckos [51,52,55], but likely also toads, small mammals and several arthropods, which increases the probability of introductions of multiple species even with few trees. Evidence for an anthropogenic introduction of the Italian wall lizards *Podarcis sicula* from southern Italy to Spain through the olive trees trade has been reported by Valdeón et al. [51] and Rivera et al. [52] for La Rioja and Sant Celoni populations respectively. The finding of numerous species of alien reptiles in the nursery gardens of the Balearic Islands provides a further evidence for this scenario.

Thus olive tree trade emerges as a modern vector for bioinvasions across the Mediterranean of a wide spectrum of alien species. Further studies covering all the Mediterranean Basin are need to uncover the magnitude and pattern of this invasion pathway, its conservation impact, and the eventual management strategies at transnational level.

### Current and future suitability of Balearic Islands for aliens snakes

As inferred from the current occupation of their native continental ranges, the Balearic Island seems of limited habitat suitability for all the four introduced species. The fact that a more relaxed threshold would predict these snake occurrences (S2 Fig.), further suggests that most of Balearic territory is still climatically suboptimal for these snake species. Nevertheless, when comparing the models’ projection with the known presence points, it becomes evident that despite this limited habitat suitability all the study species are widely distributed. Moreover, all these recently introduced species are currently spreading in the Archipelago [19].

The lack of complete congruence between the predictions and the observed records recommends a cautious interpretation of modelling results. There are three possible, not mutually exclusive, causes for the currently wider distribution of invasive snakes than predicted by the models: (i) a low performance of the models due to the use of only climatic variables and coarse spatial resolution; (ii) the favourable conditions found by these species on islands respect to the continent due to the absence of predators, parasites and competitors, or the availability of different food resources and empty niches in islands ecosystems; and (iii) finally the establishment of a source-sink patterns [56,57]. According to this latter hypothesis, the wide distribution of invasive species can be simply due to the constant release of individuals in different islands’ localities (sink) from the native range (source). Certainly, the number of adult individuals of alien snakes observed in the Balearic islands has been increasing year by year (BSD-SPEGB; MRD-COFIB; J. A. Mateo personal observation) suggesting a continual importation of snakes. However, for all the introduced species, Álvarez et al. [19] reported evidence of reproduction in the Balearic Islands- i.e. the observation of pregnant females, hatchings, and immature snakes. Therefore, the large number of snakes observed in the Balearic and their wide distribution is not merely due to continuous importation of snakes, but also to *in loco* recruitment of individuals and perhaps enhanced by the tendency of young individuals to disperse (e.g. [58]). The evidence of reproduction suggests that factors such as available habitat and food resources may be important for the future distribution and expansion of the alien snakes in these islands (see also [59–64]).

Forecasting the future, all the scenarios tested predicted an increased suitability for most of the island territory for the alien snakes (with the exception of *M*. *mauritanicus*). These results suggest that climate change might improve the environmental conditions for these invasive species. Indeed, recent studies demonstrated that climate change is already increasing the activity period and benefiting the survival and reproduction of *M*. *monspessulanus* [65–67] and *H*. *hippocrepis* [68] in the Iberian Peninsula. Therefore, the invasiveness of all snake species is expected to increase and an increase of their impact on native biota can be the anticipated.

### Conservation implications

The introduction of snakes in the Balearic Islands is expected to have negative impacts on the native biota. The four introduced species are effective predators and, according to the foraging ecology of these species in their native areas, the main impact is presumed on native lizards, amphibians, and birds (many of which are endemic and already endangered by other factors) [12]. Among other potential impacts on native fauna, alien snakes may be the vector for the introduction of new parasites. This could be particularly straightforward in the case of blood parasites since those carried by Mediterranean snakes seems to have generally low host specificity [69]. Conservation measures should be taken with a maximum urgency.

The Convention on Biological Diversity (2002) adopted the principle that the prevention of biological invasions is the priority. Only when prevention fails, early detection, rapid response, and possible eradication of invasive species should be applied [5]. The long-term management would be only the last option. In the case of Balearic Islands, suitable measures to apply depend on the timing and pathways of arrival of alien species.

Species introduced historically, like *M*. *mauritanicus* and *R*. *scalaris* in Menorca, are already widespread and naturalized during several centuries with stable reproductive contingents. Thus, the eradication for these species is not possible. However, the prevention of secondary dispersal of these species to new areas of the archipelago where they are still not present is essential. It is especially important to prevent the introduction of the snakes in the surrounding islets of Ibiza, Menorca and Mallorca and Cabrera, where Balearic endemic species such as *Podarcis lilfordi* and *P*. *pityusensis* still persist [12]. Of course this guidance apply also for those species recently introduced in the main Balearic Islands (Ibiza, Menorca, and Mallorca). Several measures can be applied to prevent further introductions within and across the Balearic archipelago: (i) inspection and quarantine of arriving goods, containers, baggage and vessels; (ii) control and monitoring of importation of trees or other plants; (iii) methodical inspection on maritime cargo. Preventive actions may also benefit from studies aimed at evaluating the risk of invasion across the Balearic Islands associated with current touristic and commercial activities.

In cases where prevention would reveal ineffective, the early detection and the rapid eradication of invasive species should be pursued. For such programs to be successful, a specific educational effort for the general public is needed. Currently, Balearic regional authorities have designed a snake eradication program which they are already implementing in Ibiza and Formentera. These islands deserve the maximum priority since they have never harboured snakes. The first actions of eradication in Ibiza have included the involvement of the general public and snake collection combining traps and dogs search in the main areas of snake sightings [70]. Despite the considerable amount of economic and human resources devoted to the eradication, the outcome of this program is still highly uncertain, because in this case the snake invasion already moved forward from the earlier stages as indicated by the occurrence of cases of reproduction [70]. On the other hand, these actions would certainly help to restrict the spread of the invasion within the islands and to keep the density of the alien snakes as low as possible, thus minimizing their impact and their probability of spread across the archipelago.

In conclusion, in this study the combination of molecular and ecological tools with chronogeonemic data on the introduced snakes allowed dissecting the main pathways and dynamic of the process of biological invasions. This knowledge will be crucial for implementing preventive strategies to avoid further introductions within the main islands and the arrival of the alien snakes to the satellite islets which still host much native biodiversity. Moreover, the identification of the olive trees trade as a powerful vector of alien species claims for the attention of conservationist to apply the required preventive measures across the Mediterranean areas potentially threatened by such new waves of biological invasions.

## Supporting Information

S1 Fig Jackknife analyses. Jackknife re-sampling results of the training and test gain and of AUC values for all the four species used in the study.
(PDF)

S2 Fig Habitat suitability models with logistic threshold. Habitat suitability models for the present and for the future (2020, 2050 and 2080) of all the species in study, with the balance training omission, predicted area and threshold value “logistic threshold” (a lower threshold compared to the one used on the principal study).
(PDF)

S3 Fig MESS analysis. (Left) Year and scenario corresponding to the results. (Centre) MESS results: areas in red have one or more environmental variables outside the present range in the training data. (B) MoD results, showing the most dissimilar variable.
(PDF)

S1 Table Genetic Data. Information on locality, gene fragment and GenBank accession number of each sequence used in the genetic analysis.
(PDF)
