# Supplementary material for: Snakes on the Balearic Islands: An Invasion Tale with Implications for Native Biodiversity Conservation
Source: PLoS One. 2015 Apr 8;10(4):e0121026. doi: 10.1371/journal.pone.0121026 (PMC4390158; doi:10.1371/journal.pone.0121026)

**S1 Figure: Jack-knife analysis.** Jack-knife re-sampling results of the training and test gain and of AUC values for all the four species in study.

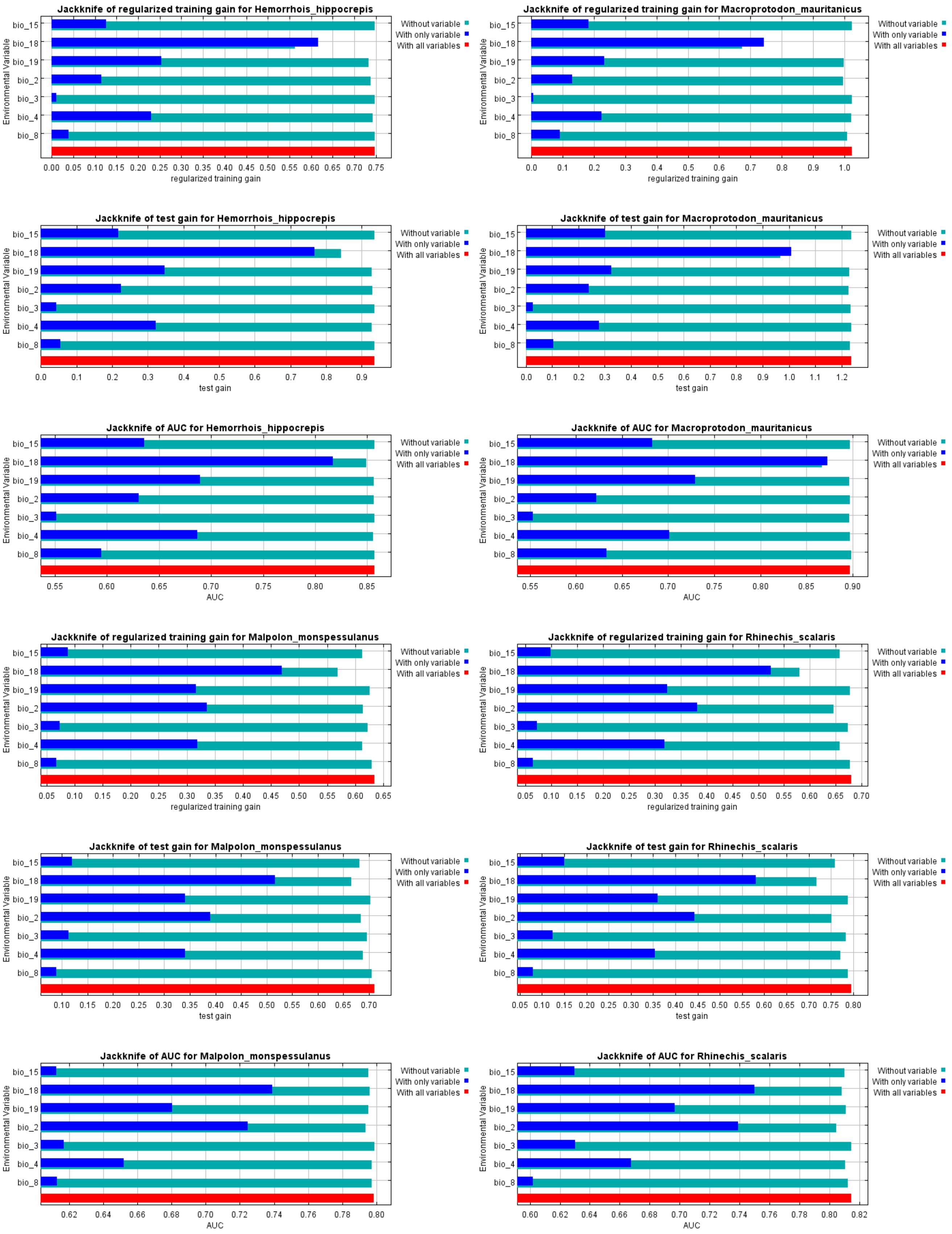

Supplement: S1 Fig — Jackknife re-sampling results of the training and test gain and of AUC values for all the four species used in the study. (PDF) [file pone.0121026.s001.pdf]
