# Supplementary material for: Snakes on the Balearic Islands: An Invasion Tale with Implications for Native Biodiversity Conservation
Source: PLoS One. 2015 Apr 8;10(4):e0121026. doi: 10.1371/journal.pone.0121026 (PMC4390158; doi:10.1371/journal.pone.0121026)
Supplement: S2 Fig — Habitat suitability models for the present and for the future (2020, 2050 and 2080) of all the species in study, with the balance training omission, predicted area and threshold value “logistic threshold” (a lower threshold compared to the one used on the principal study). (PDF) [file pone.0121026.s002.pdf]

S2 Figure: Habitat suitability models with logistic threshold. Habitat suitability models for the present and for the future (2020, 2050 and 2080) of all the species in study, with the balance training omission, predicted area and threshold value “logistic threshold” (a lower threshold compared to the one used on the principal study).

	Hemorrhois hippocrepis	*Macroprotodon mauritanicus*	*Malpolon monspessulanus*	*Rhinechis scalaris*
Present	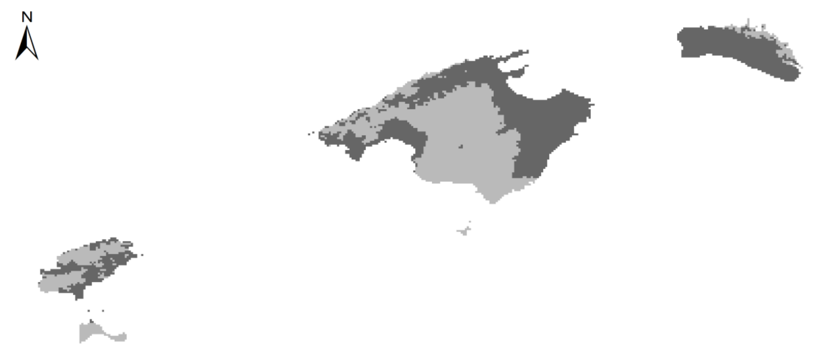	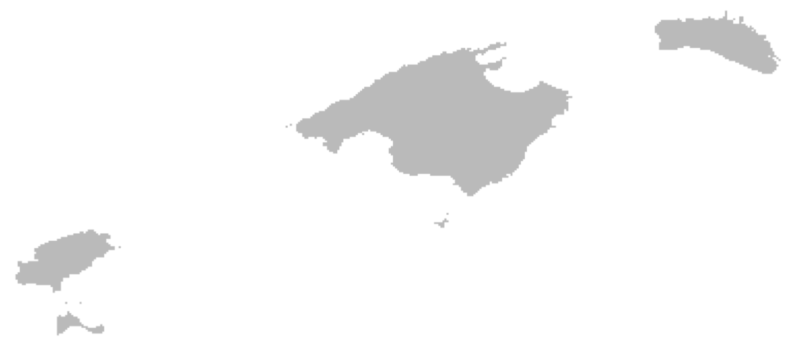	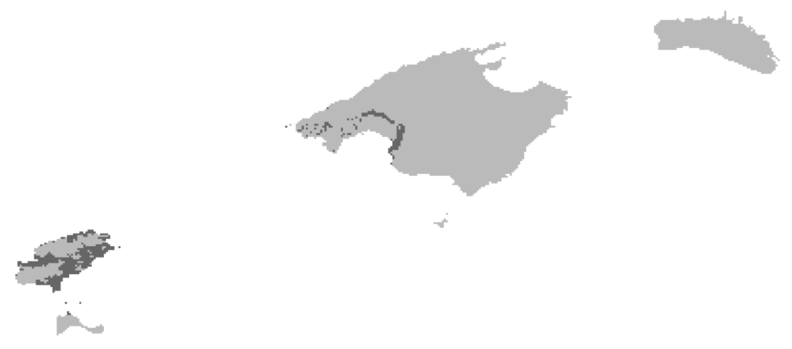	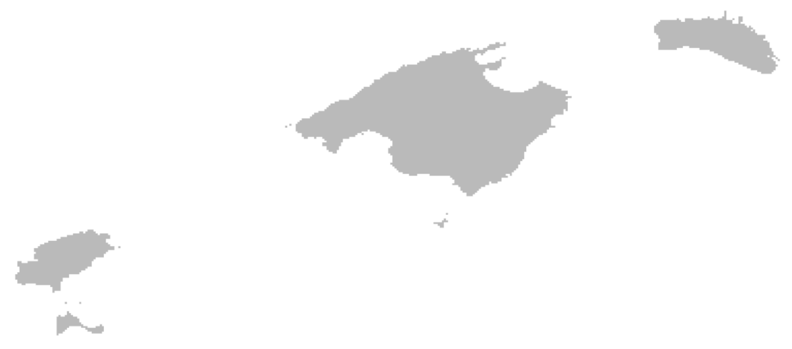
2020	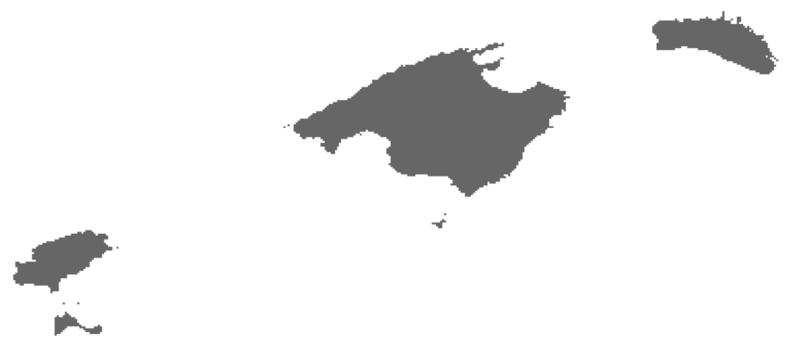	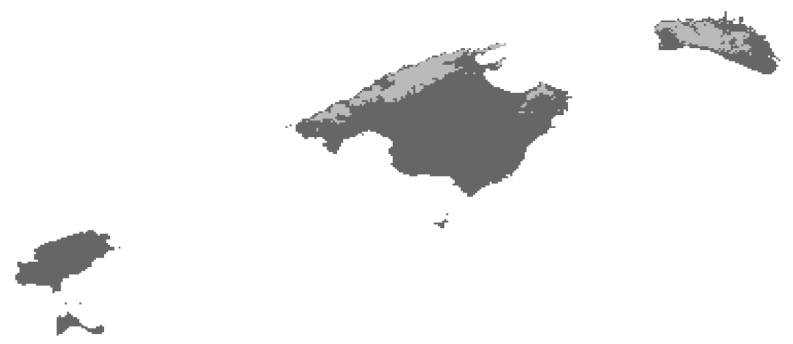	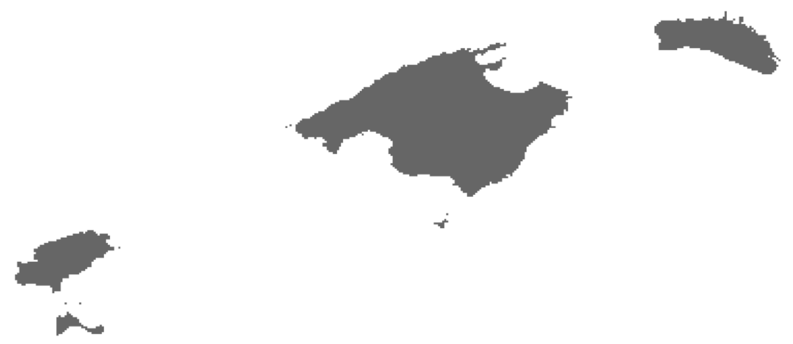	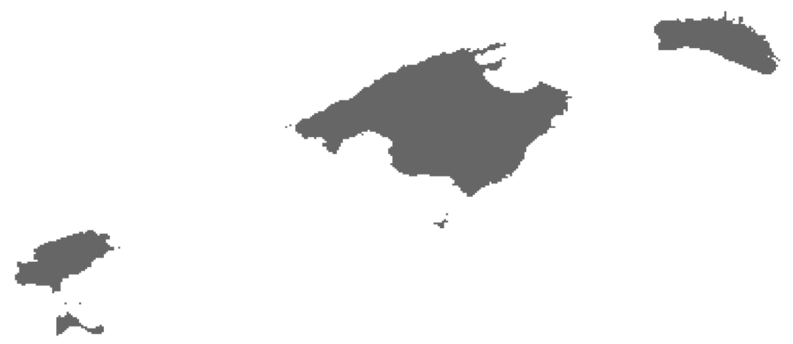
2050	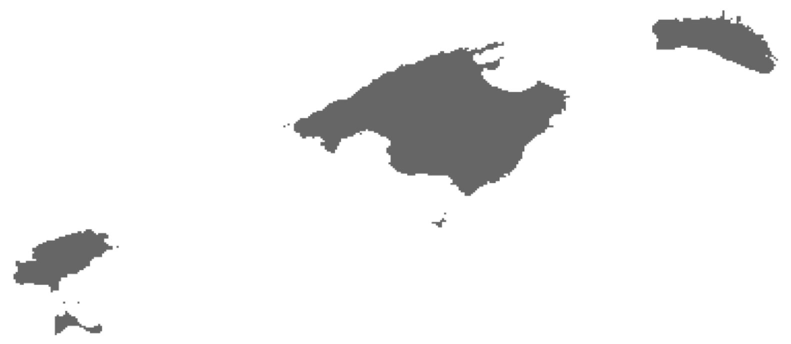	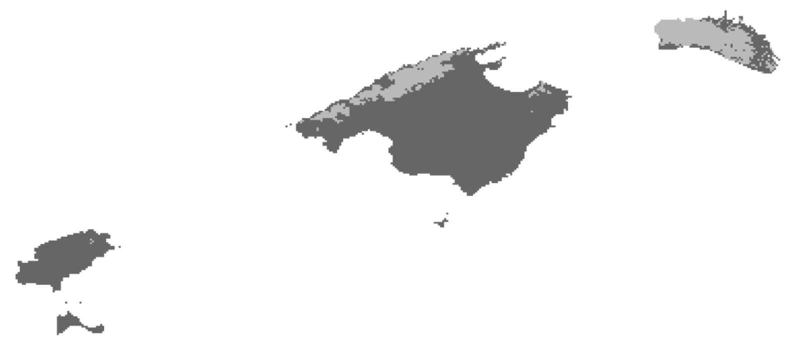	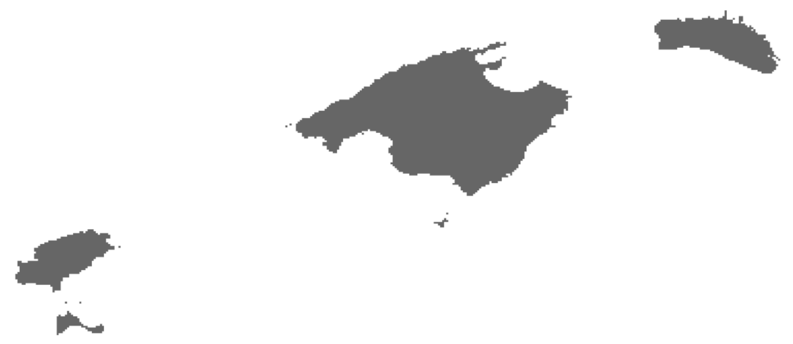	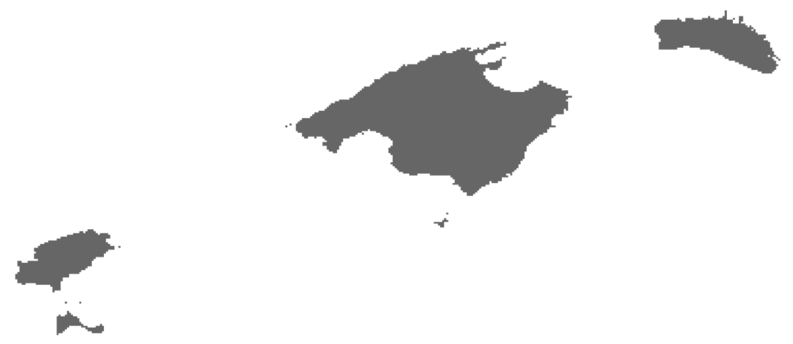
2080	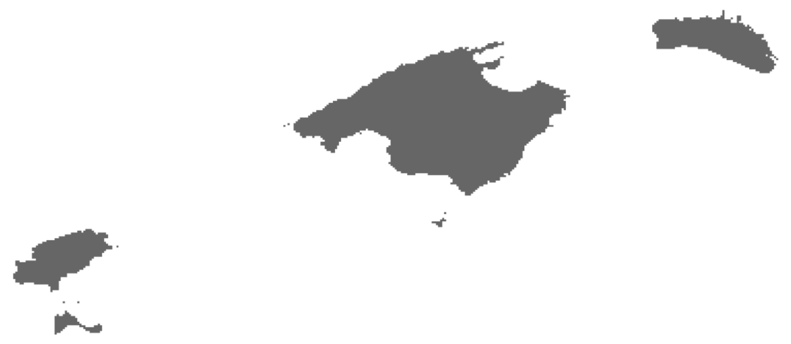	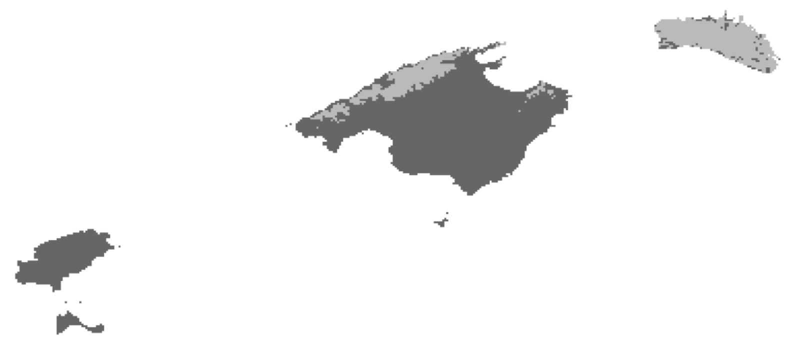	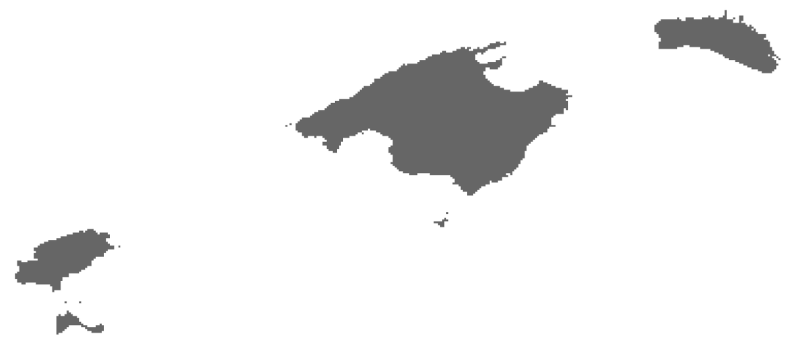	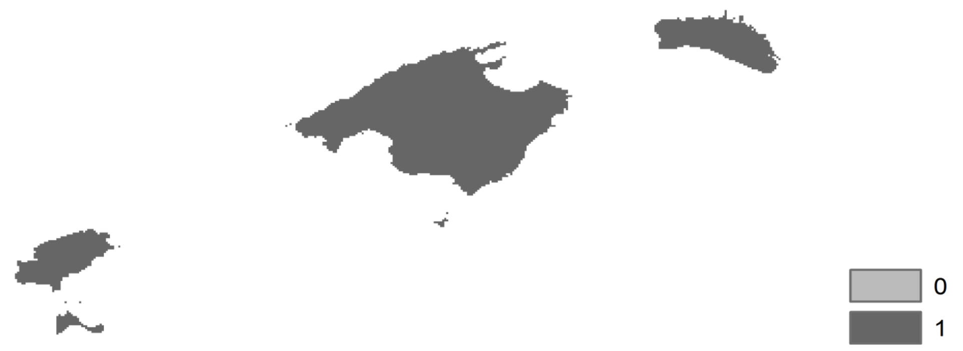
