# Supplementary material for: Snakes on the Balearic Islands: An Invasion Tale with Implications for Native Biodiversity Conservation
Source: PLoS One. 2015 Apr 8;10(4):e0121026. doi: 10.1371/journal.pone.0121026 (PMC4390158; doi:10.1371/journal.pone.0121026)
Supplement: S3 Fig — (Left) Year and scenario corresponding to the results. (Centre) MESS results: areas in red have one or more environmental variables outside the present range in the training data. (B) MoD results, showing the most dissimilar variable. (PDF) [file pone.0121026.s003.pdf]

S3 Figure: MESS analysis results. On the left are shown Multivariate Environmental Similarity Surfaces results (MESS): areas in red have one or more environmental variables outside the present range in the training data. On the right, MoD results showing the most dissimilar variable.

Hemorrhois hippocrepis		
Year/Scenario	MESS	MoD
Present	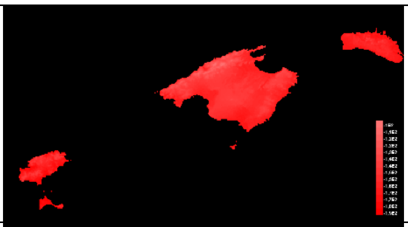	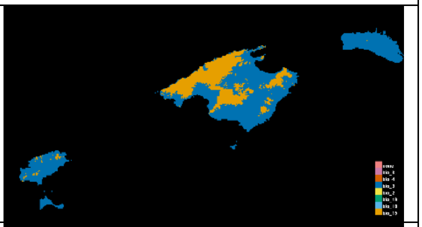
A1b_cccma_2020	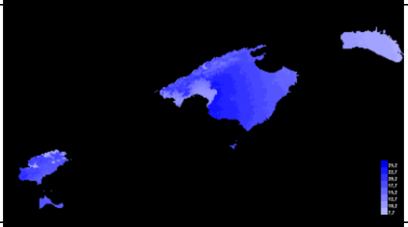	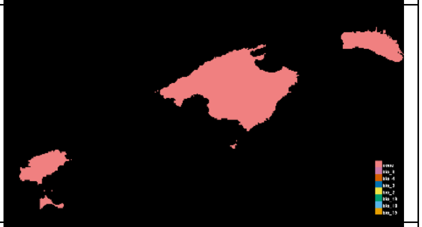
A1b_cccma_2050	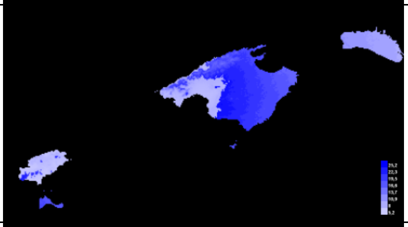	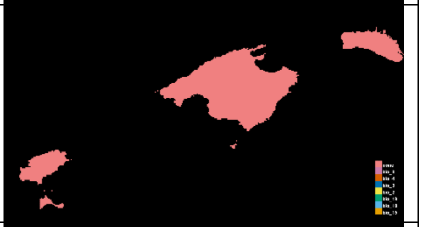
A1b_cccma_2080	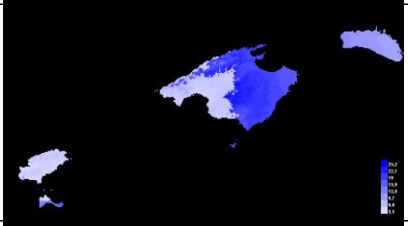	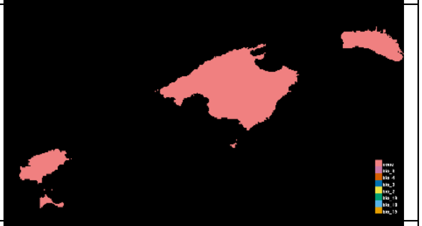
A1b_csiro_2020	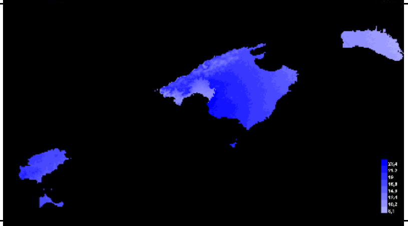	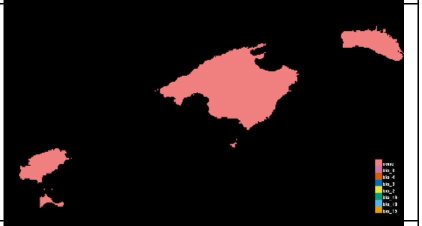
A1b_csiro_2050	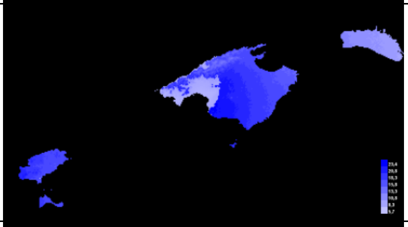	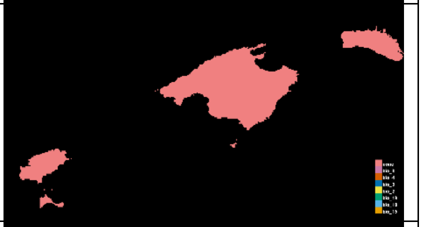
A1b_csiro_2080	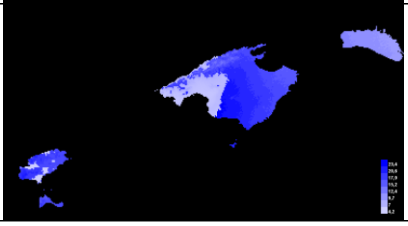	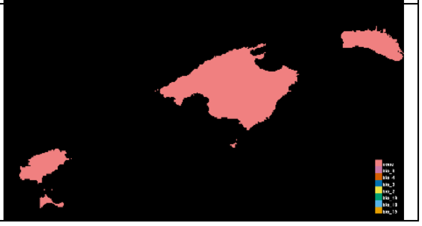

A1b_hadcm3_2020	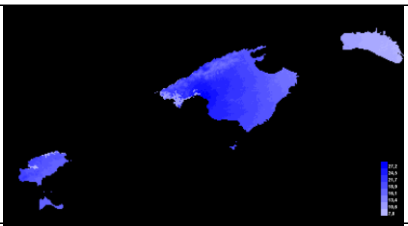	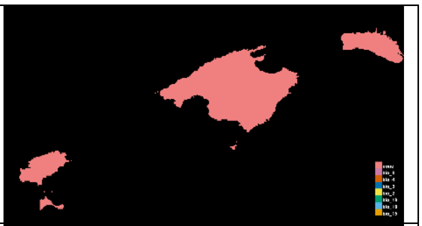
A1b_hadcm3_2050	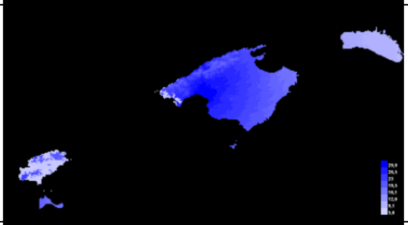	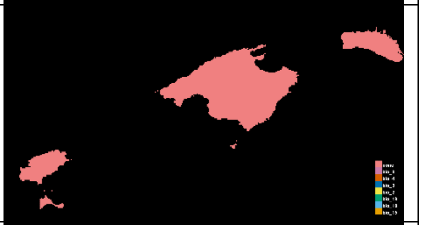
A2a_cccma_2020	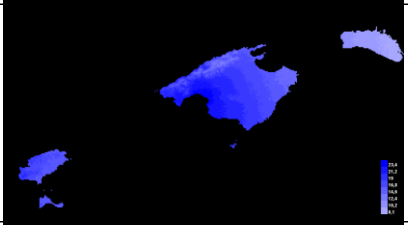	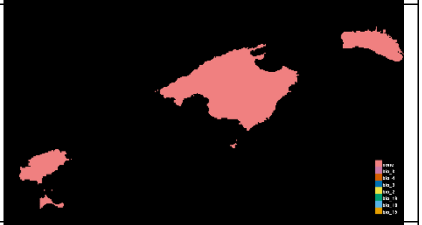
A2a_cccma_2050	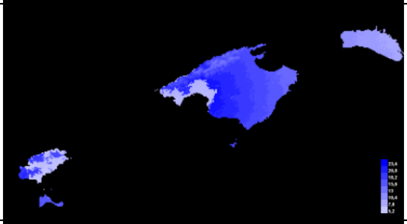	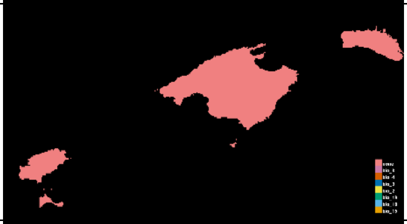
A2a_cccma_2080	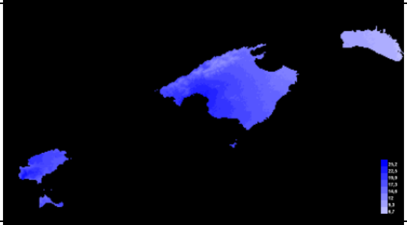	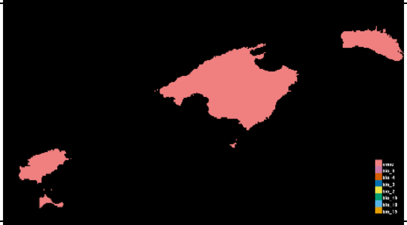
A2a_csiro_2020	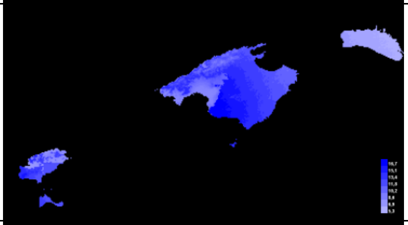	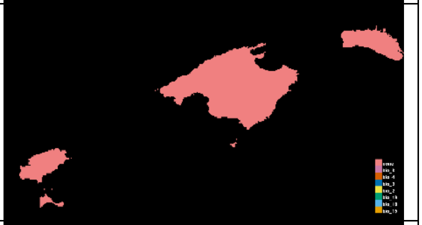
A2a_csiro_2050	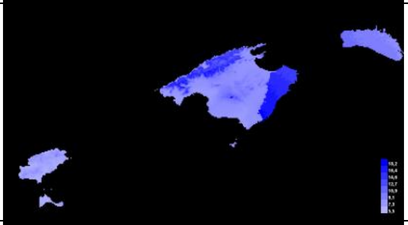	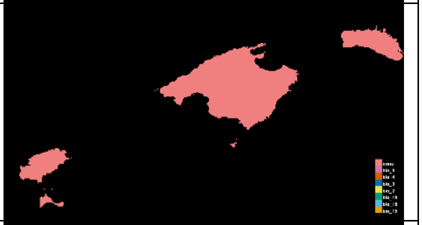
A2a_csiro_2080	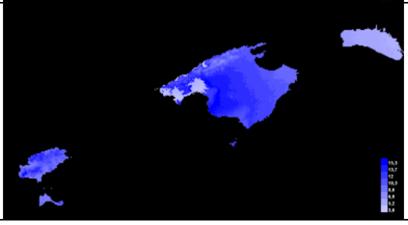	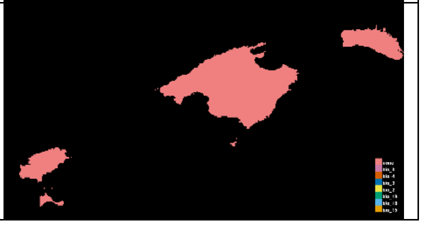

A2a_hadcm3_2020	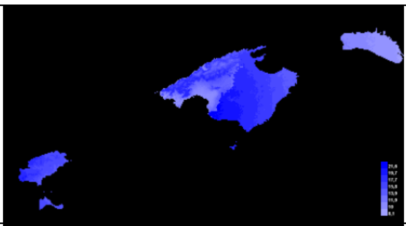	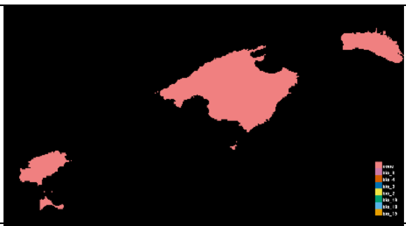
A2a_hadcm3_2050	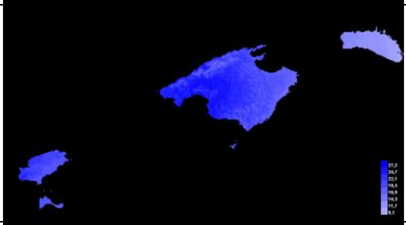	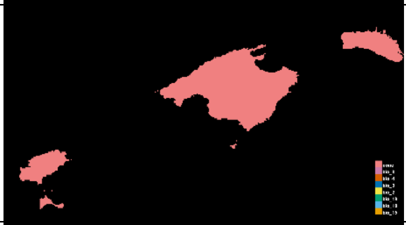
A2a_hadcm3_2080	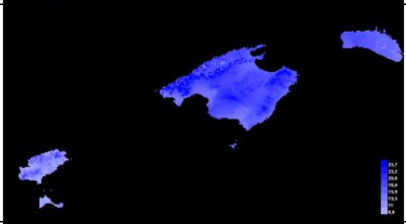	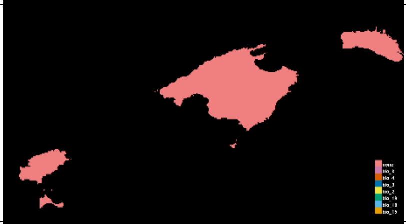
B2a_cccma_2020	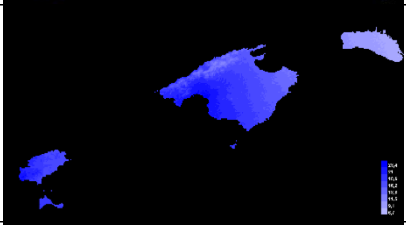	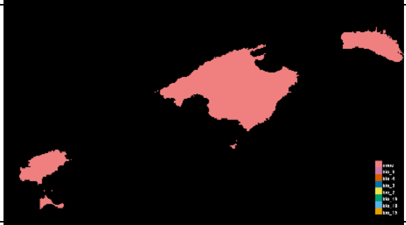
B2a_cccma_2050	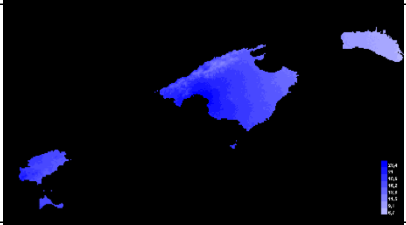	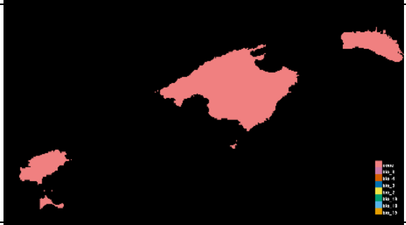
B2a_cccma_2080	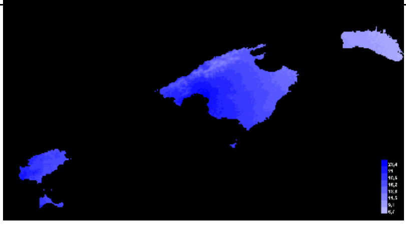	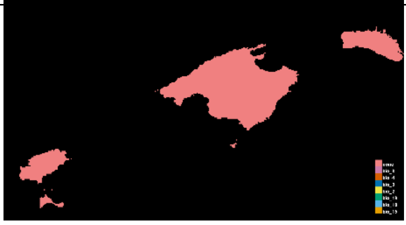
B2a_csiro_2020	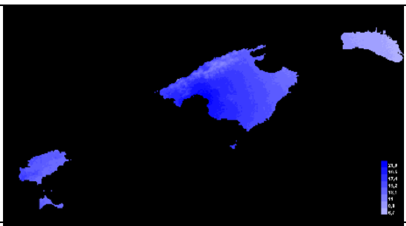	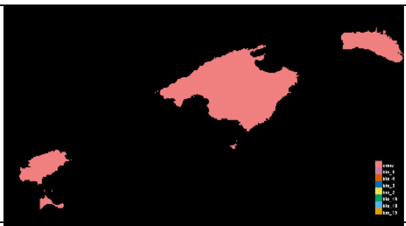
B2a_csiro_2050	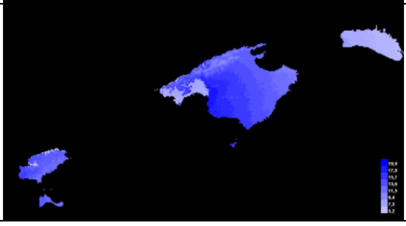	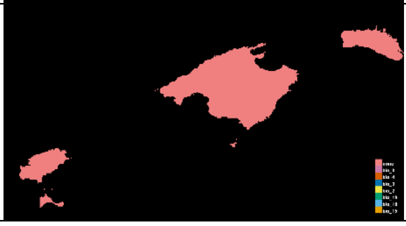

B2a_csiro_2080	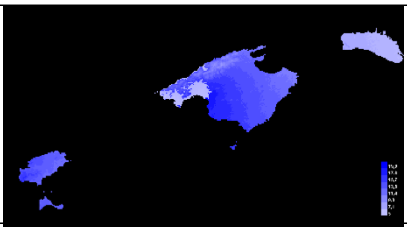	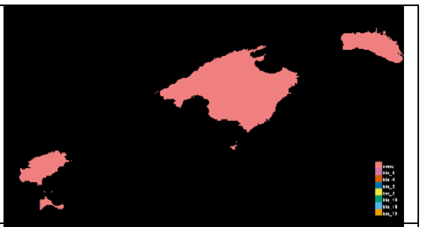
B2a_hadcm3_2020	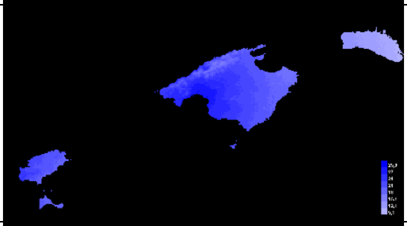	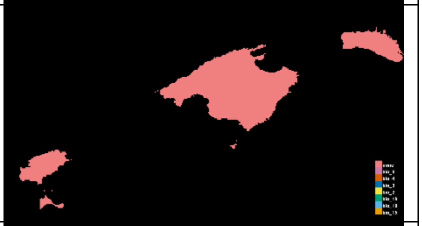
B2a_hadcm3_2050	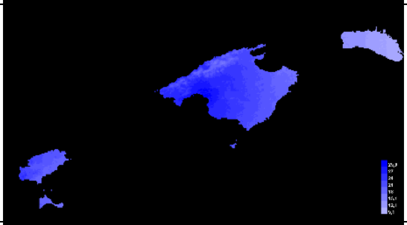	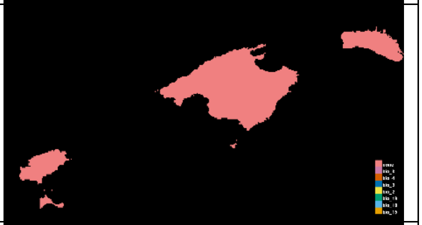
B2a_hadcm3_2080	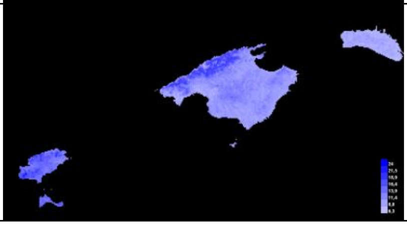	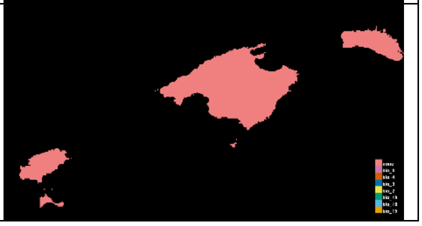

Macroprotodon sp.		
Year/Scenario	MESS	MoD
Present	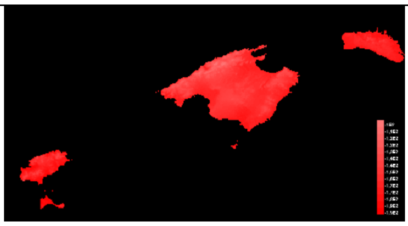	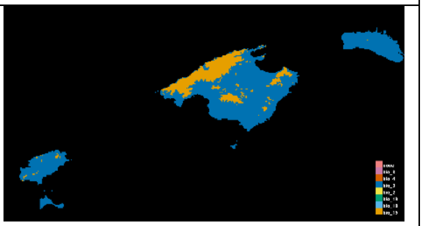
A1b_cccma_2020	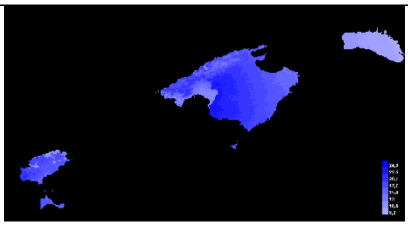	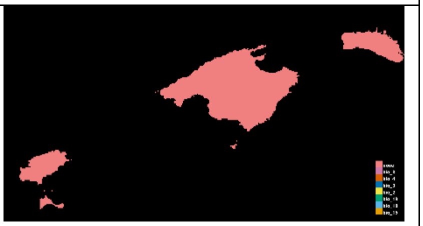
A1b_cccma_2050	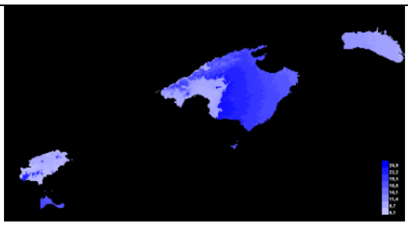	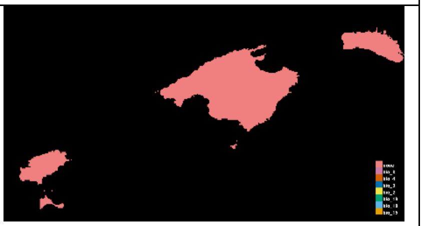
A1b_cccma_2080	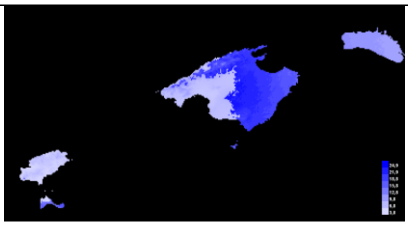	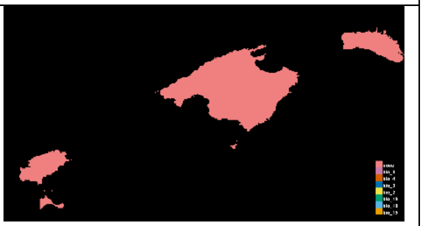
A1b_csiro_2020	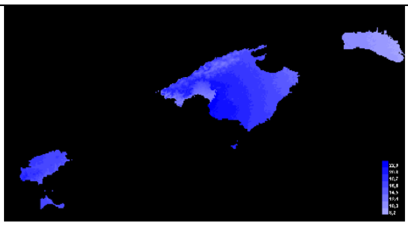	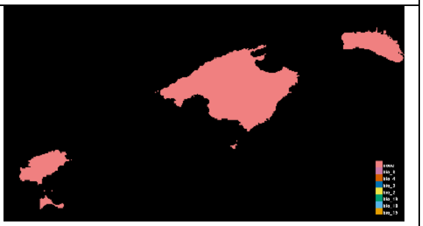
A1b_csiro_2050	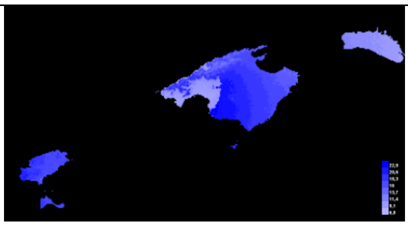	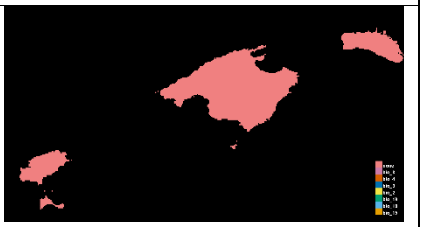
A1b_csiro_2080	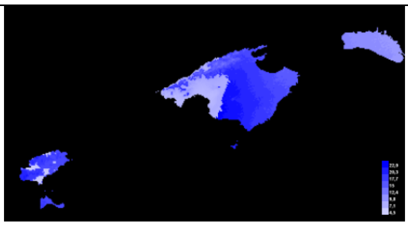	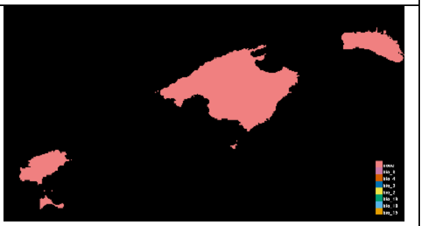

A1b_hadcm3_2020	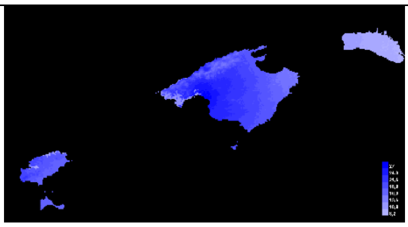	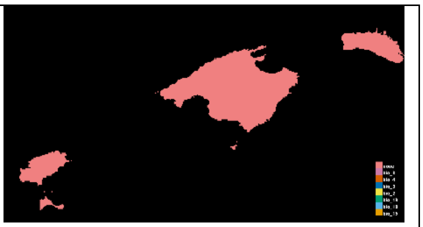
A1b_hadcm3_2050	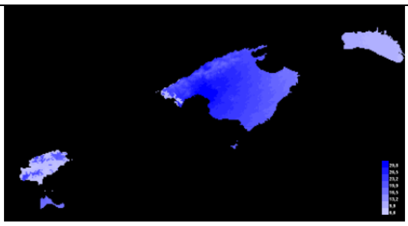	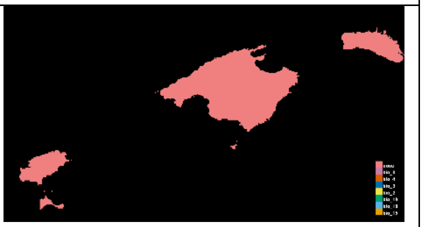
A2a_cccma_2020	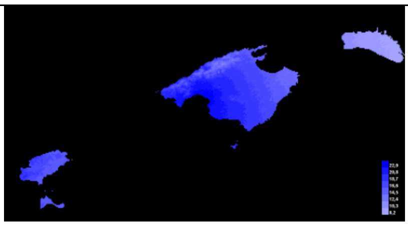	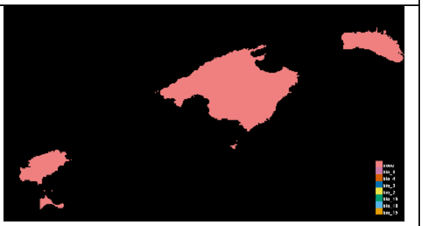
A2a_cccma_2050	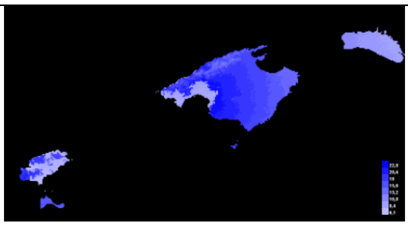	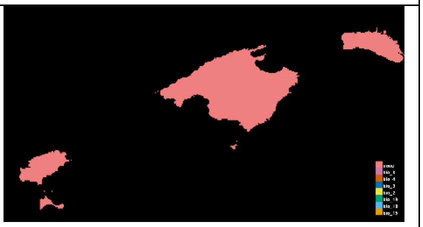
A2a_cccma_2080	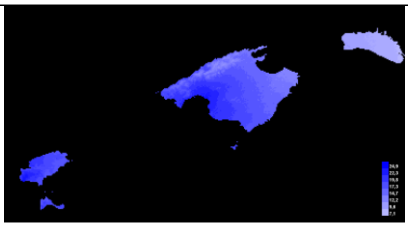	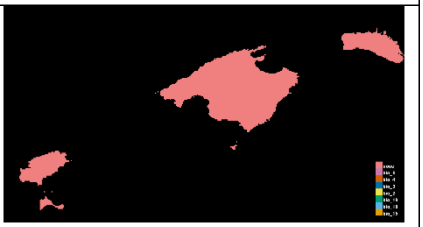
A2a_csiro_2020	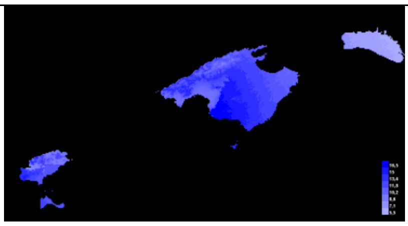	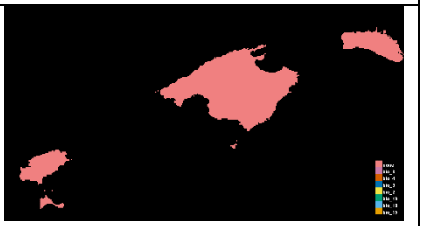
A2a_csiro_2050	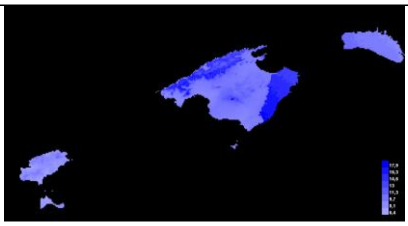	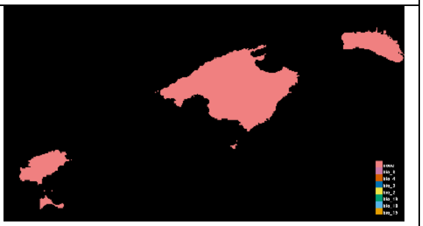

A2a_csiro_2080	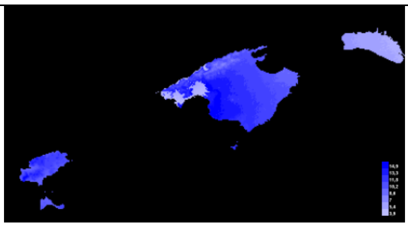	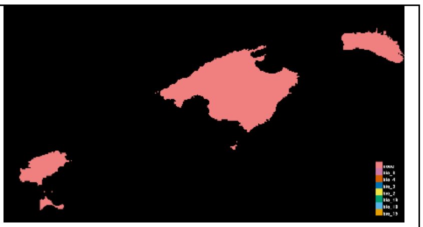
A2a_hadcm3_2020	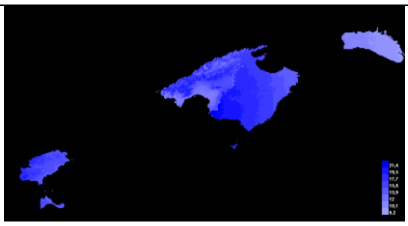	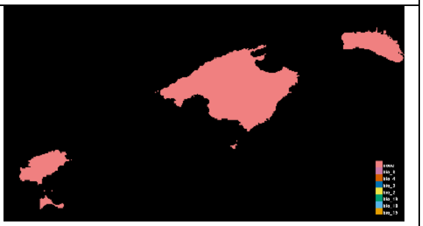
A2a_hadcm3_2050	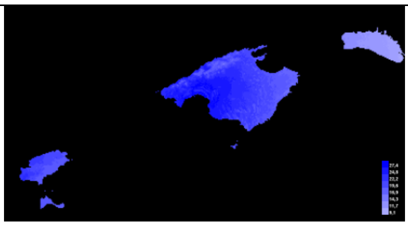	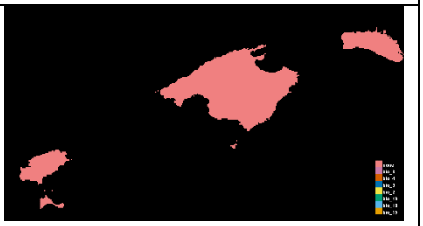
A2a_hadcm3_2080	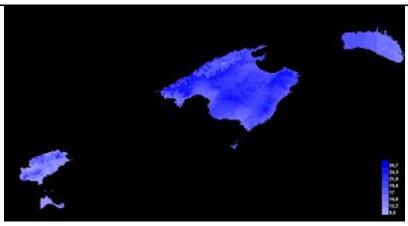	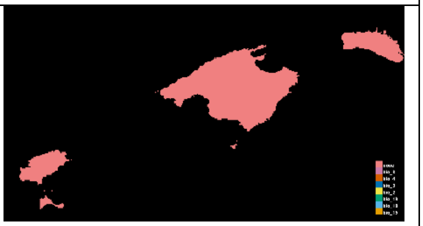
B2a_cccma_2020	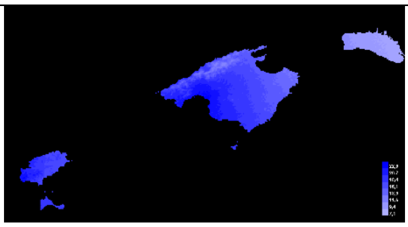	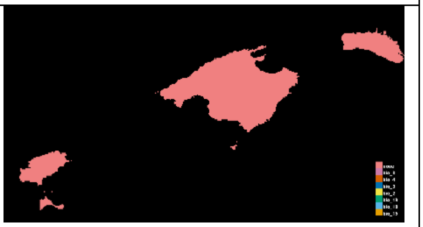
B2a_cccma_2050	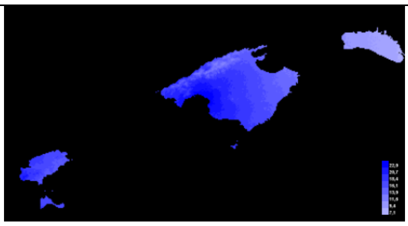	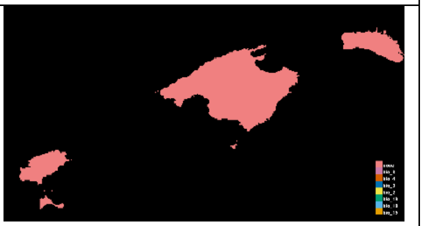
B2a_cccma_2080	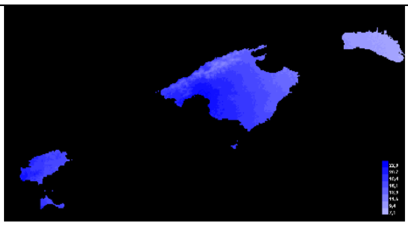	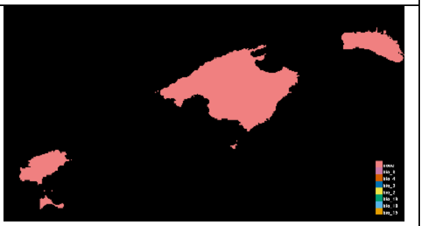

B2a_csiro_2020	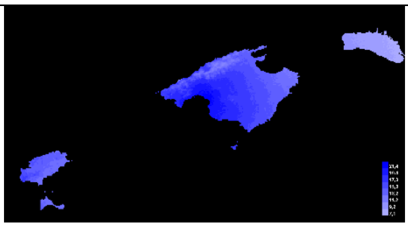	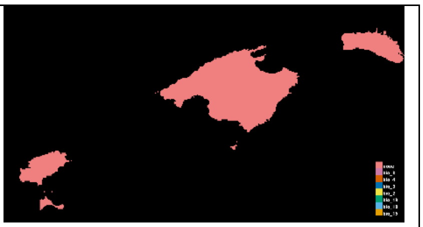
B2a_csiro_2050	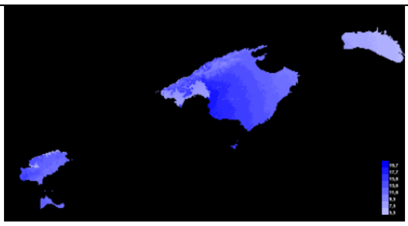	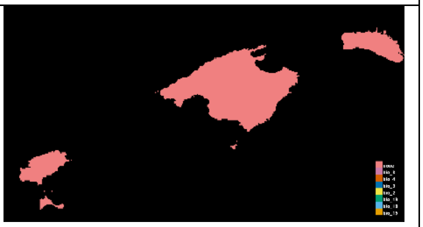
B2a_csiro_2080	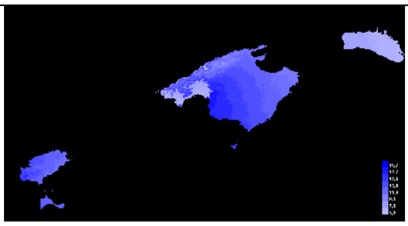	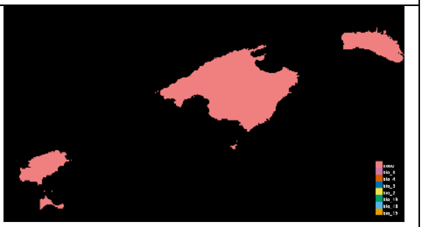
B2a_hadcm3_2020	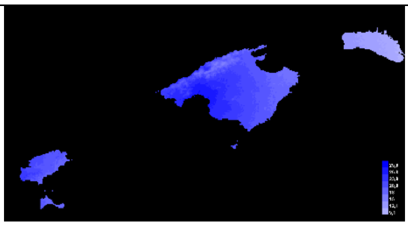	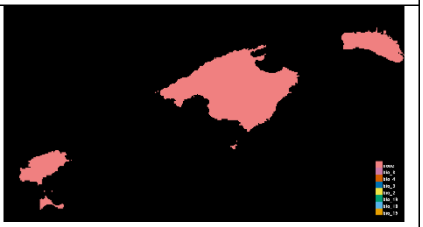
B2a_hadcm3_2050	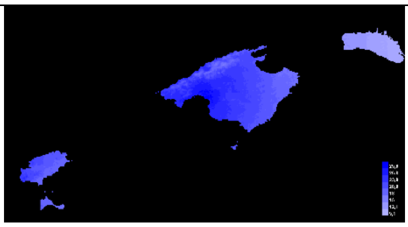	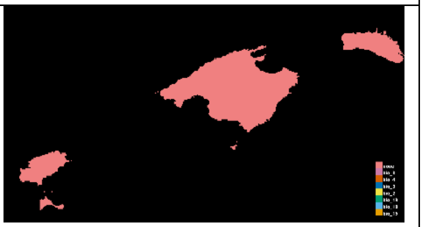
B2a_hadcm3_2080	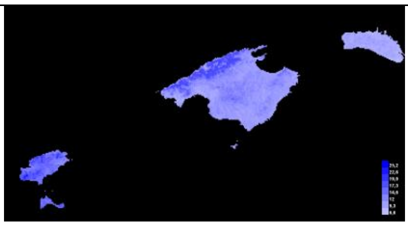	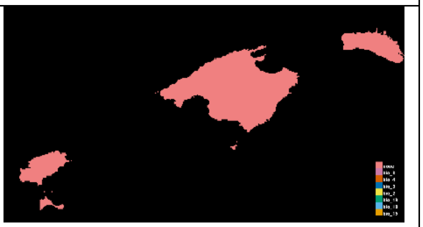

Malpolon monspessulanus		
Year/Scenario	MESS	MoD
Present	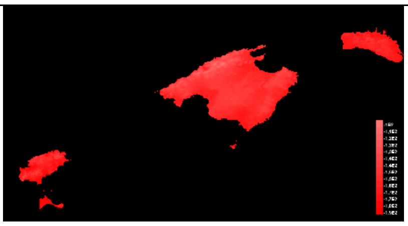	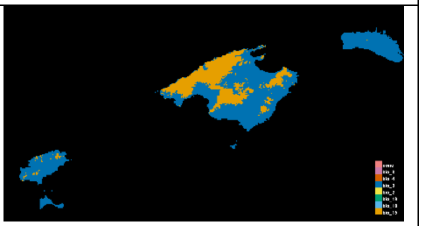
A1b_cccma_2020	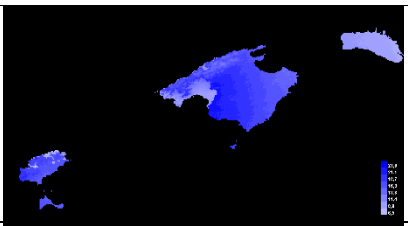	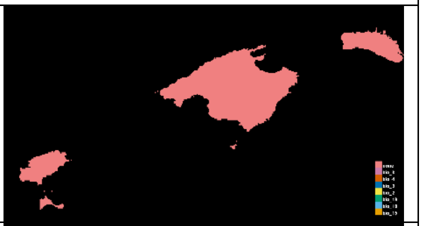
A1b_cccma_2050	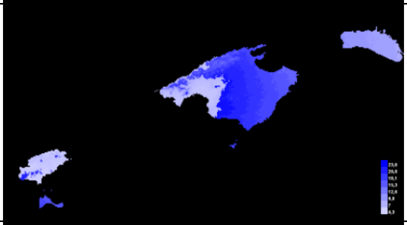	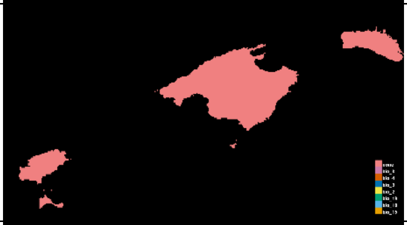
A1b_cccma_2080	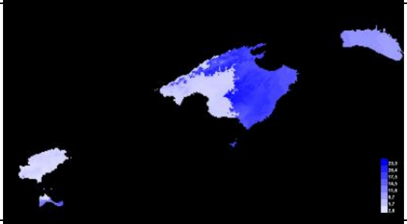	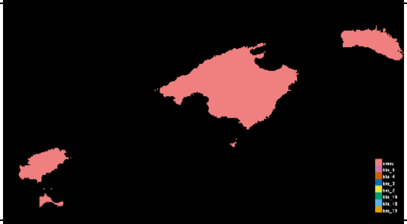
A1b_csiro_2020	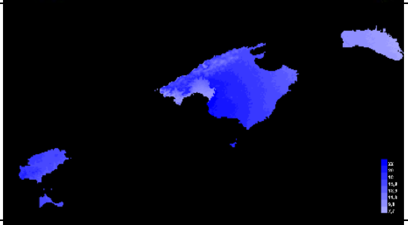	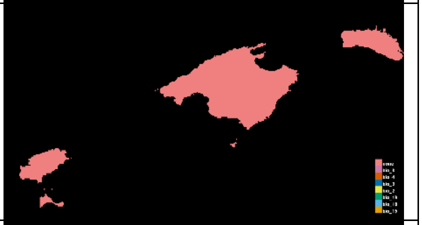
A1b_csiro_2050	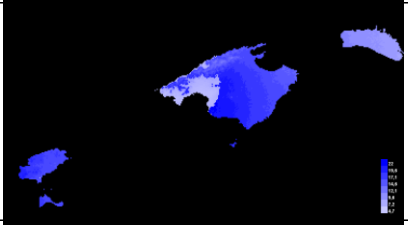	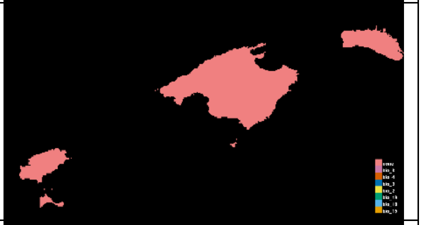
A1b_csiro_2080	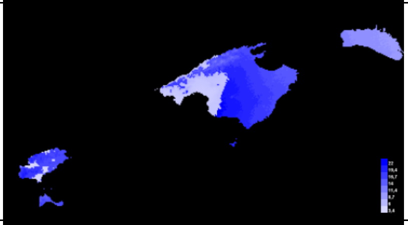	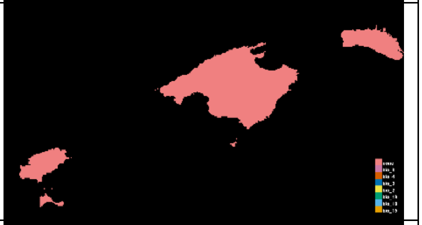
A1b_hadcm3_2020	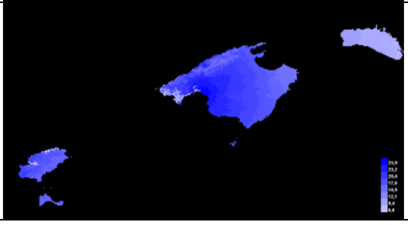	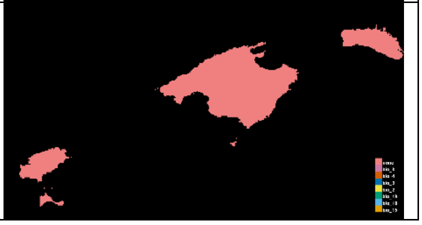

A1b_hadcm3_2050	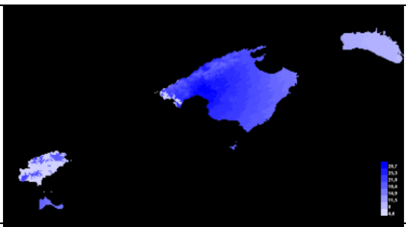	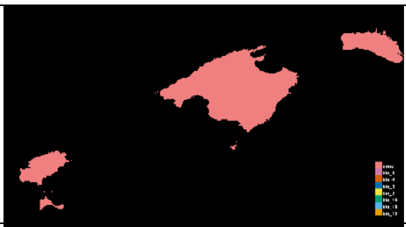
A2a_cccma_2020	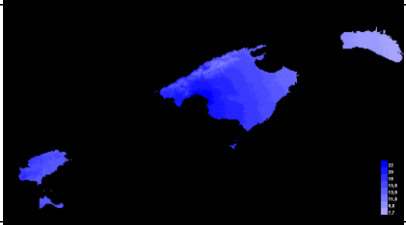	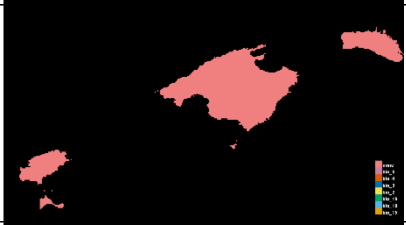
A2a_cccma_2050	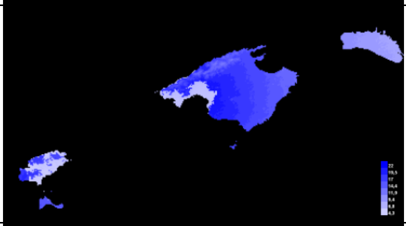	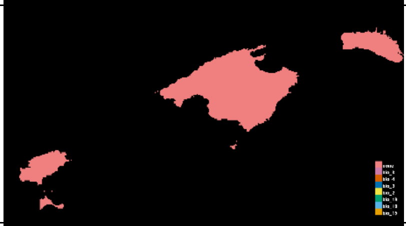
A2a_cccma_2080	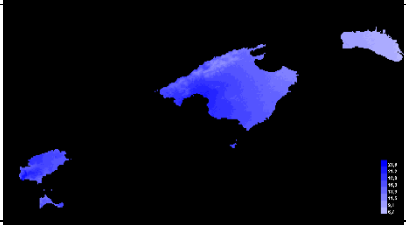	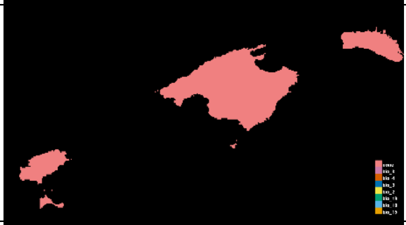
A2a_csiro_2020	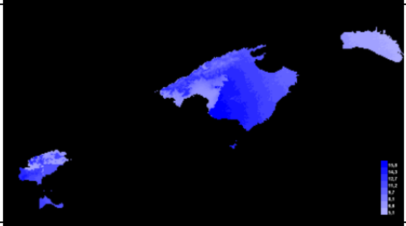	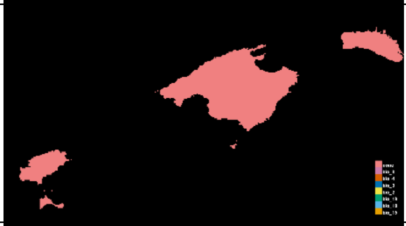
A2a_csiro_2050	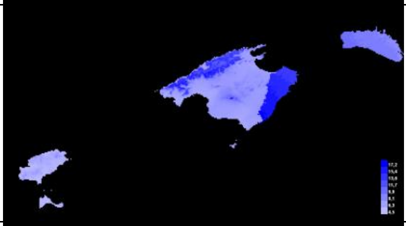	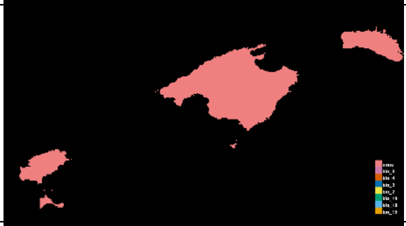
A2a_csiro_2080	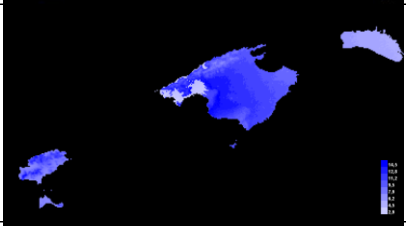	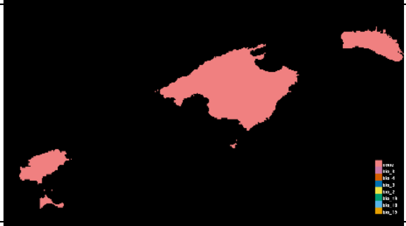
A2a_hadcm3_2020	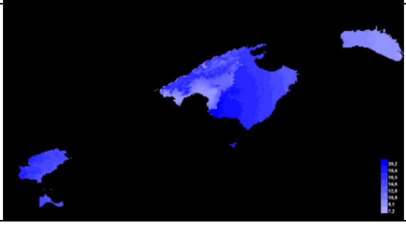	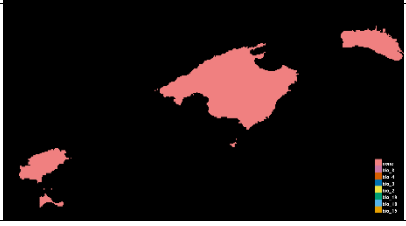

A2a_hadcm3_2050	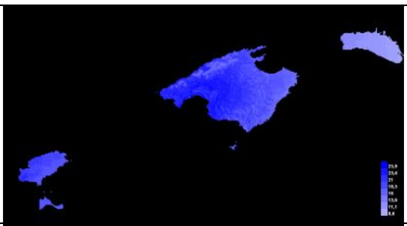	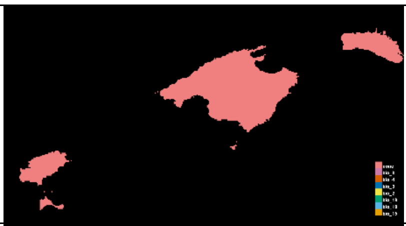
A2a_hadcm3_2080	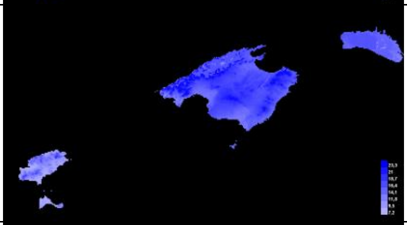	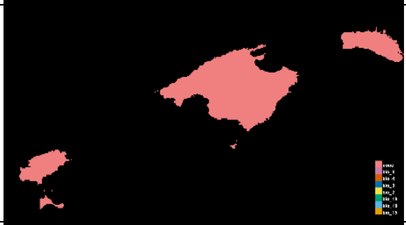
B2a_cccma_2020	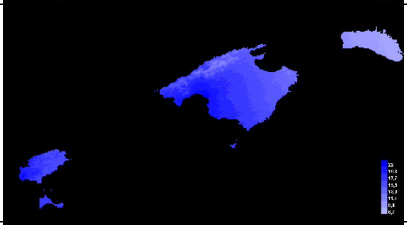	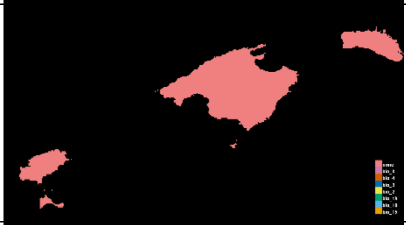
B2a_cccma_2050	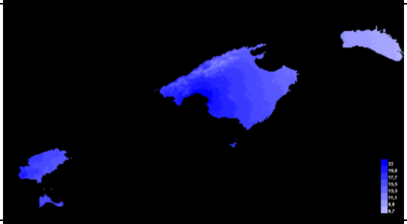	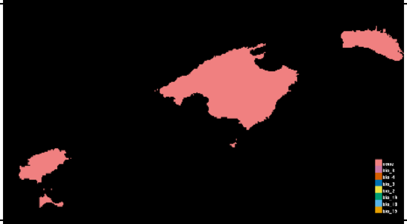
B2a_cccma_2080	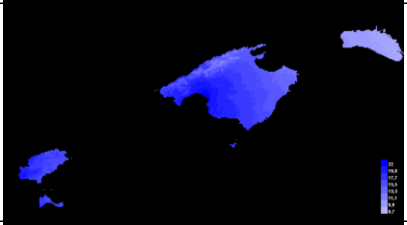	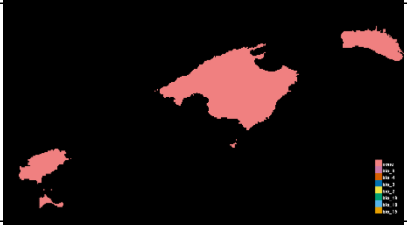
B2a_csiro_2020	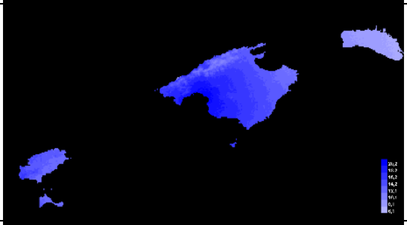	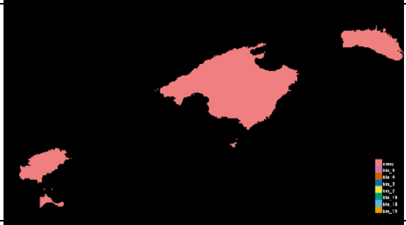
B2a_csiro_2050	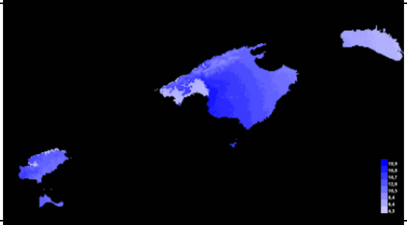	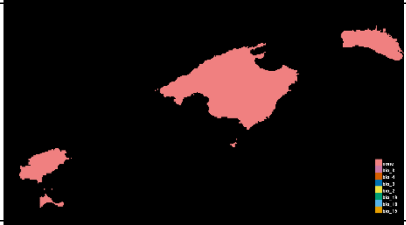
B2a_csiro_2080	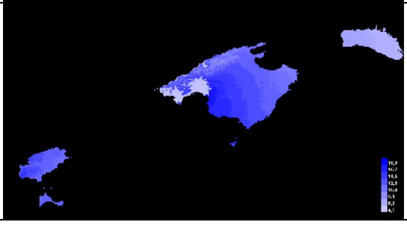	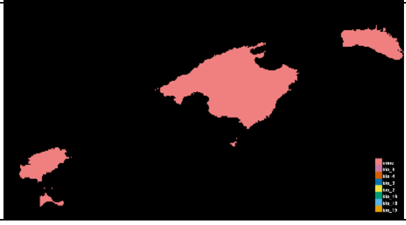

B2a_hadcm3_2020	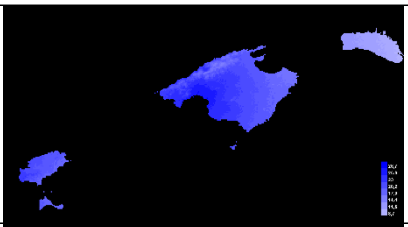	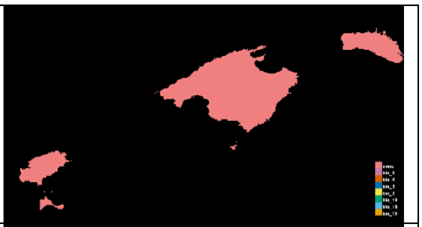
B2a_hadcm3_2050	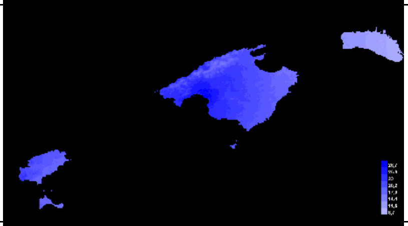	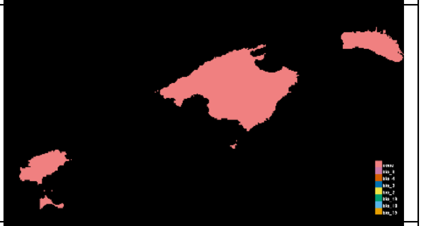
B2a_hadcm3_2080	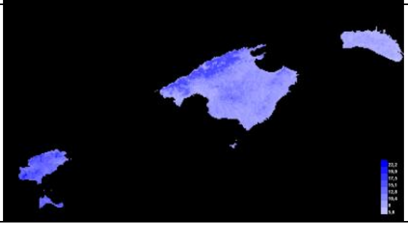	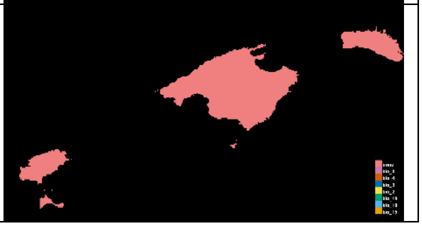

Rhinechis scalaris		
Year/Scenario	MESS	MoD
Present	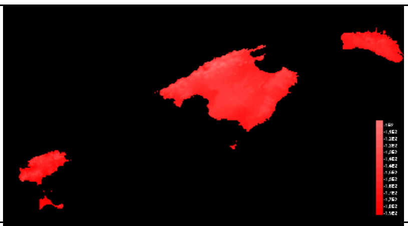	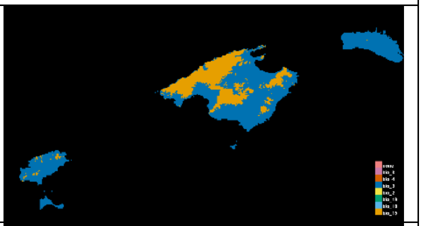
A1b_cccma_2020	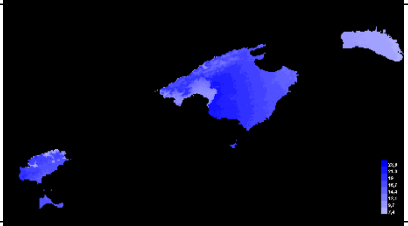	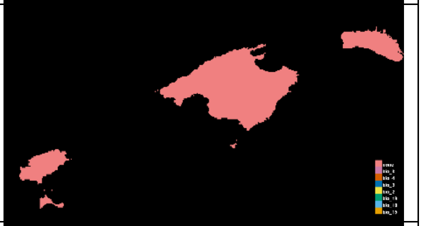
A1b_cccma_2050	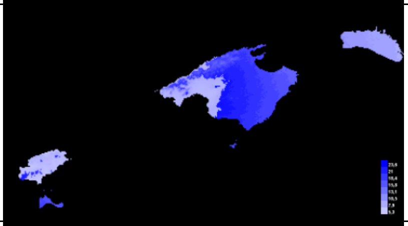	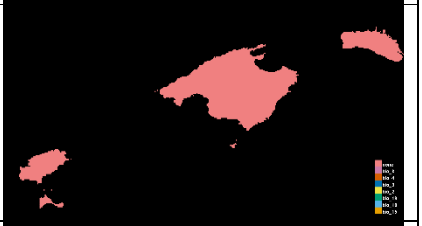
A1b_cccma_2080	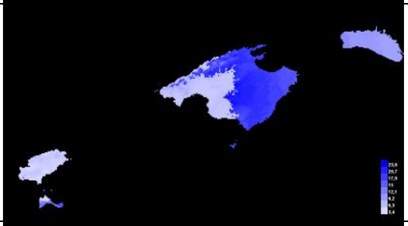	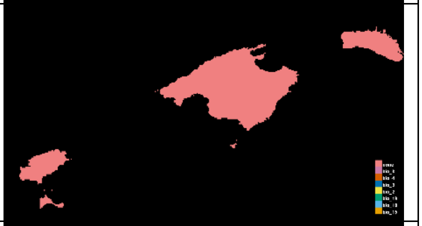
A1b_csiro_2020	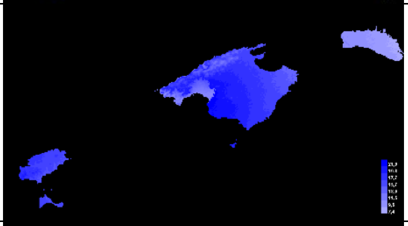	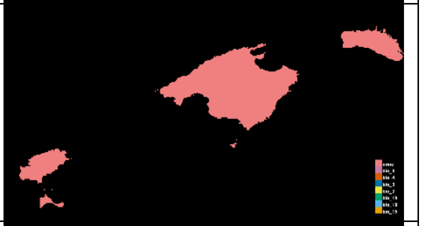
A1b_csiro_2050	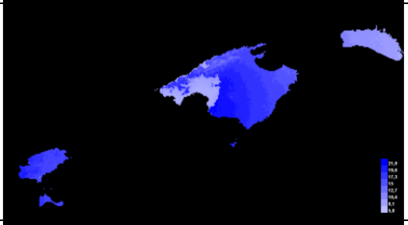	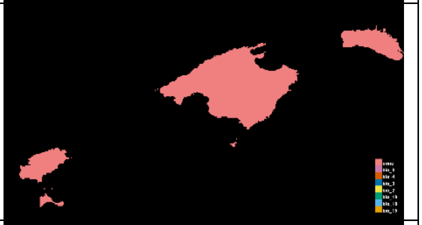
A1b_csiro_2080	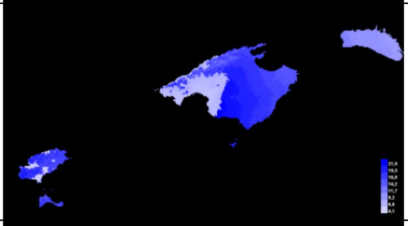	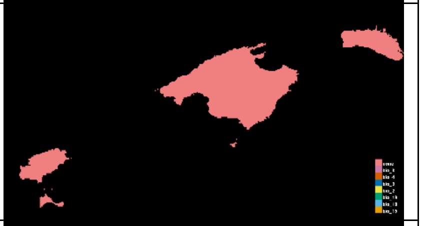
A1b_hadcm3_2020	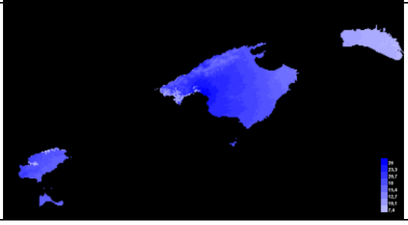	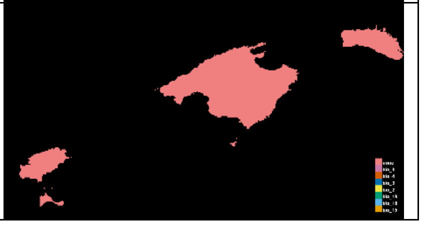

A1b_hadcm3_2050	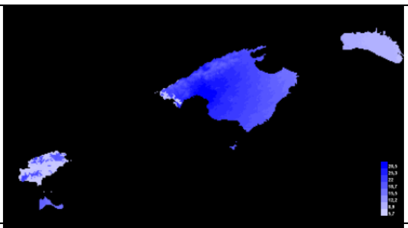	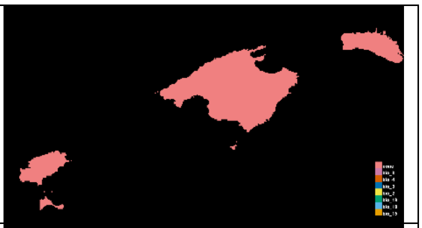
A2a_cccma_2020	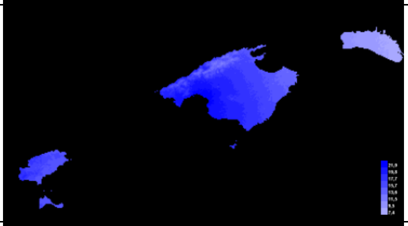	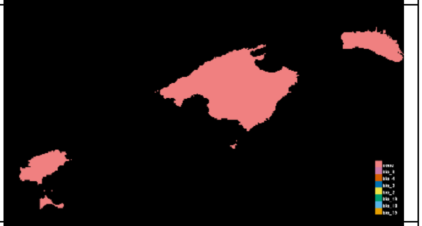
A2a_cccma_2050	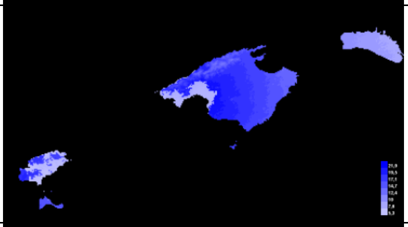	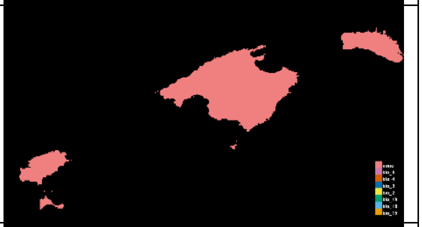
A2a_cccma_2080	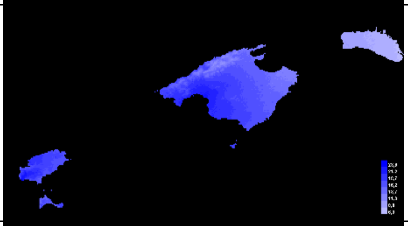	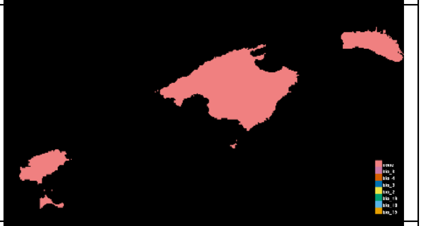
A2a_csiro_2020	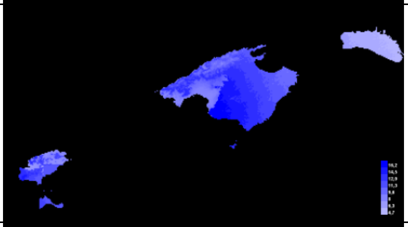	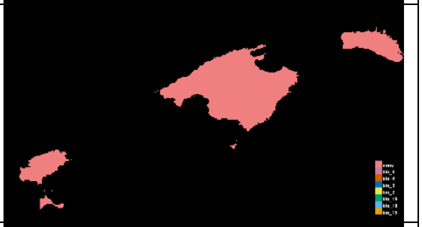
A2a_csiro_2050	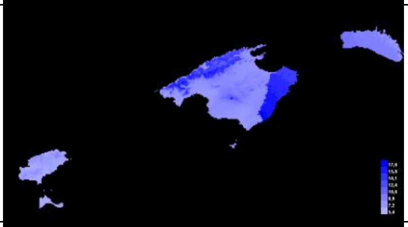	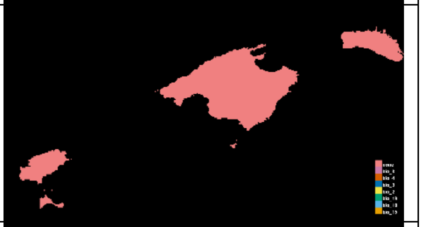
A2a_csiro_2080	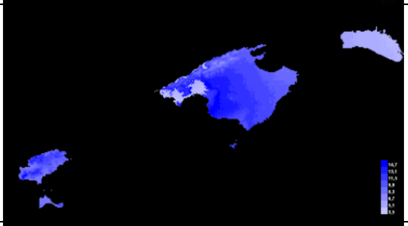	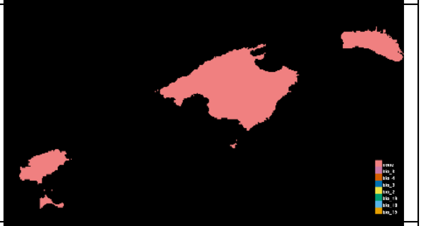
A2a_hadcm3_2020	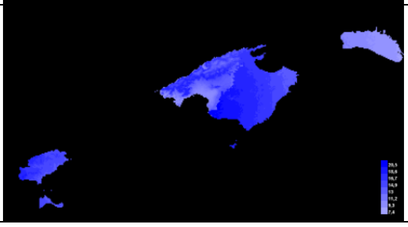	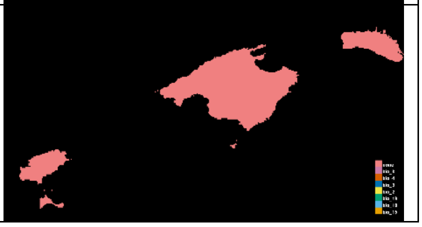

A2a_hadcm3_2050	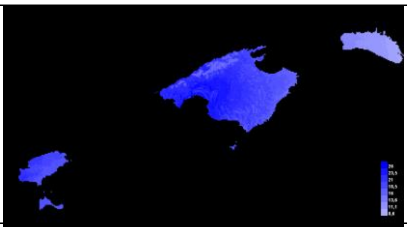	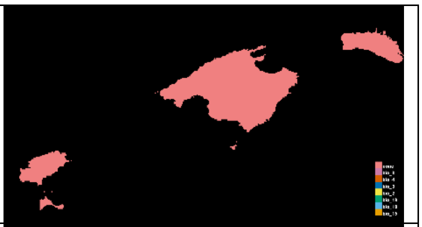
A2a_hadcm3_2080	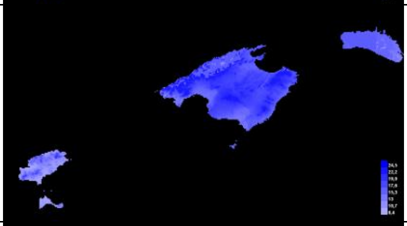	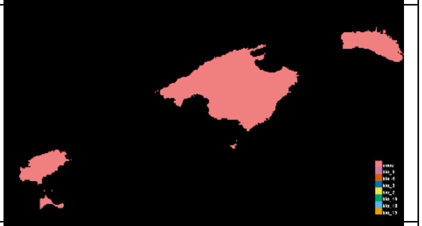
B2a_cccma_2020	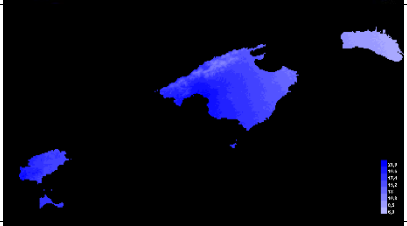	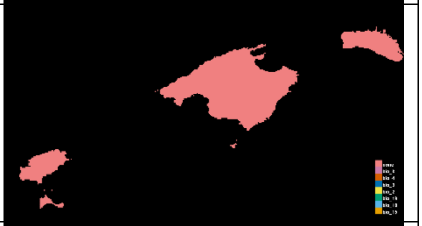
B2a_cccma_2050	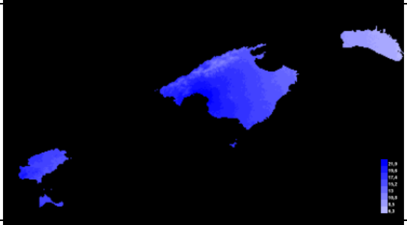	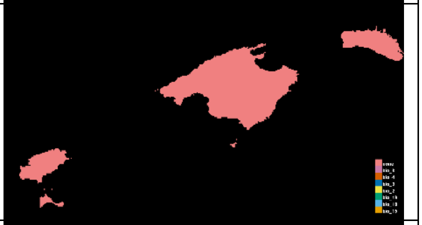
B2a_cccma_2080	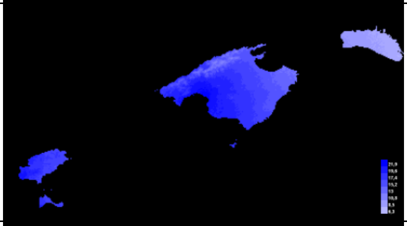	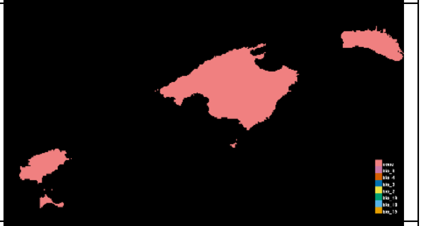
B2a_csiro_2020	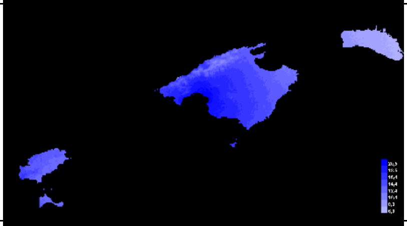	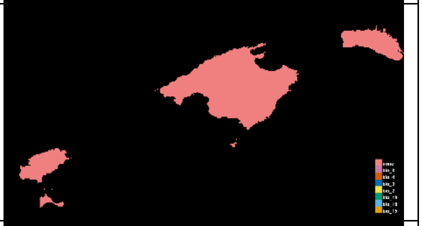
B2a_csiro_2050	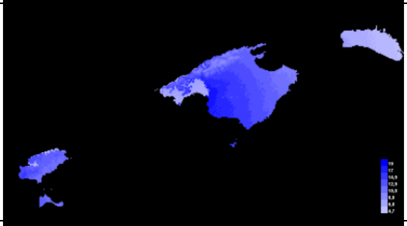	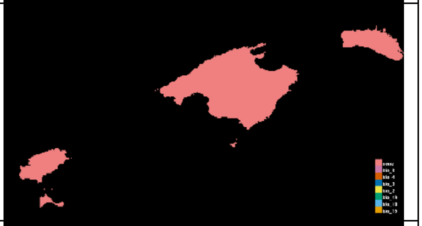
B2a_csiro_2080	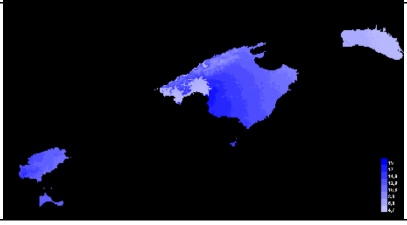	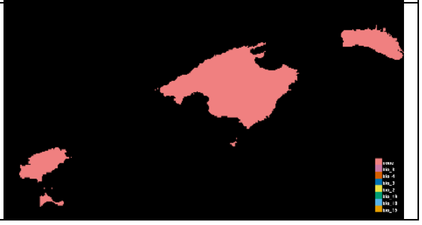

B2a_hadcm3_2020	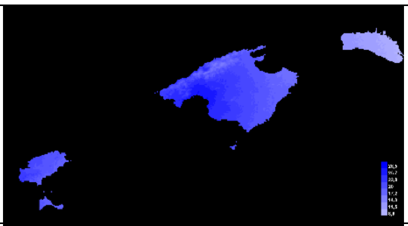	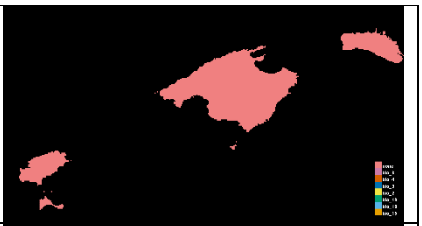
B2a_hadcm3_2050	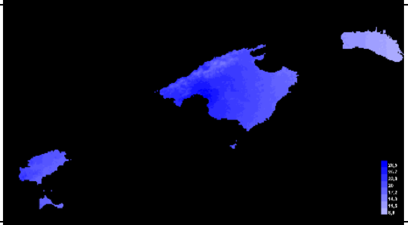	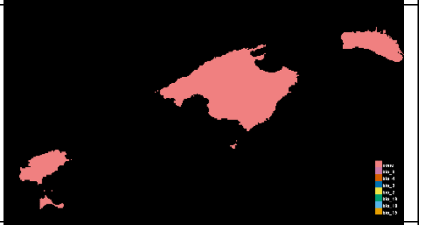
B2a_hadcm3_2080	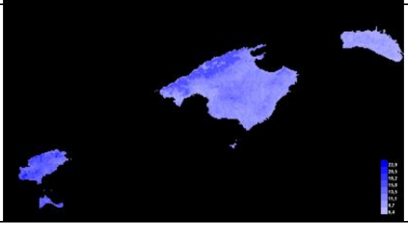	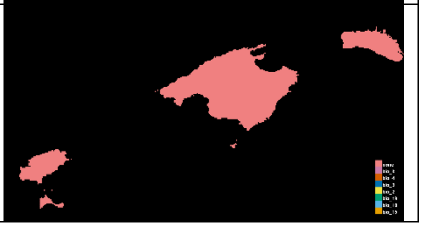
