# Supplementary material for: Snakes on the Balearic Islands: An Invasion Tale with Implications for Native Biodiversity Conservation
Source: PLoS One. 2015 Apr 8;10(4):e0121026. doi: 10.1371/journal.pone.0121026 (PMC4390158; doi:10.1371/journal.pone.0121026)
Supplement: S1 Table — Information on locality, gene fragment and GenBank accession number of each sequence used in the genetic analysis. (PDF) [file pone.0121026.s004.pdf]

S1 Table: Genetic Data. Information on locality, gene fragment and GenBank accession number of each sequence used in the genetic analysis.

Species	Sample code	Locality	Gene Fragment	Genbank Accession number
Hemorrhois hippocrepis	DB9177	Capdepera, Mallorca	*cytb* , *12S*	KP036572, KP036559
Hemorrhois hippocrepis	DB9240	Capdepera, Mallorca	*cytb*	KP036585
Hemorrhois hippocrepis	DB9285	Capdepera, Mallorca	*cytb*	KP036573
Hemorrhois hippocrepis	DB10533	Capdepera, Mallorca	*cytb* , *12S*	KP036574, KP036558
Hemorrhois hippocrepis	DB11104	Capdepera, Mallorca	*cytb*	KP036580
Hemorrhois hippocrepis	DB5209	Capdepera, Mallorca	*cytb*	KP036567
Hemorrhois hippocrepis	DB5214	Capdepera, Mallorca	*cytb*	KP036569
Hemorrhois hippocrepis	DB5213	Capdepera, Mallorca	*cytb*	KP036587
Hemorrhois hippocrepis	DB5215	Capdepera, Mallorca	*cytb* , *12S*	KP036570, KP036560
Hemorrhois hippocrepis	DB11869	Capdepera, Mallorca	*cytb*	KP036583
Hemorrhois hippocrepis	DB13450	Capdepera, Mallorca	*cytb* , *12S*	KP036581, KP036557
Hemorrhois hippocrepis	DB13453	Capdepera, Mallorca	*cytb*	KP036564
Hemorrhois hippocrepis	DB9164	Artá, Mallorca	*cytb*	KP036571
Hemorrhois hippocrepis	DB5210	Artà, Mallorca	*cytb*	KP036568
Hemorrhois hippocrepis	DB13454	Magaluf,Mallorca	*cytb* , *12S*	KP036565, KP036554
Hemorrhois hippocrepis	DB13449	Calvia (Magaluf), Mallorca	*cytb* , *12S*	KP036582, KP036553
Hemorrhois hippocrepis	DB10546	Sineu, Mallorca	*cytb*	KP036584
Hemorrhois hippocrepis	DB5153	Mallorca	*cytb* , *12S*	KP036566, KP036555
Hemorrhois hippocrepis	DB5216	Mallorca	*cytb* , *12S*	KP036586, KP036556
Hemorrhois hippocrepis	DB13784	Can Toni Sa Rota,Ibiza	*cytb*	KP036577
Hemorrhois hippocrepis	DB13785	Santa Eularia, Ibiza	*cytb* , *12S*	KP036575, KP036551
Hemorrhois hippocrepis	DB13786	Santa Eularia, Ibiza	*cytb* , *12S*	KP036576, KP036550
Hemorrhois hippocrepis	DB13787	Ibiza	*cytb* , *12S*	KP036579, KP036552
Hemorrhois hippocrepis	DB13788	Sant Joan,Ibiza	*cytb*	KP036578
Hemorrhois hippocrepis	DQ451988/DQ451960	Huelva, Spain	*cytb* , *12S*	DQ451960/DQ451988
Hemorrhois hippocrepis	DQ451990/DQ451961	Cadiz, Spain	*cytb* , *12S*	DQ451961/DQ451990
Hemorrhois hippocrepis	DQ451991/DQ451962	Sevilla, Spain	*cytb* , *12S*	DQ451962/DQ451991
Hemorrhois hippocrepis	DQ451999/DQ451977	Tremp, Spain	*cytb* , *12S*	DQ451977/DQ451999
Hemorrhois hippocrepis	DQ452000/DQ451978	Malgrat del Mar, Spain	*cytb* , *12S*	DQ451978/DQ452000
Hemorrhois hippocrepis	DQ451989/DQ451954	Kahaoucha, Morocco	*cytb* , *12S*	DQ451954/DQ451989
Hemorrhois hippocrepis	DQ451992/DQ451963	Ras el Ma, Morocco	*cytb* , *12S*	DQ451963/DQ451992
Hemorrhois hippocrepis	DQ451993/DQ451965	Zinat, Morocco	*cytb* , *12S*	DQ451965/DQ451993
Hemorrhois hippocrepis	DQ451997/DQ451974	Tetouan, Morocco	*cytb* , *12S*	DQ451974/DQ451997
Hemorrhois hippocrepis	DQ451998/DQ451970	Fez, Morocco	*cytb* , *12S*	DQ451970/DQ451998
Hemorrhois hippocrepis	DQ452001/DQ451984	Azrou/Ifrane, Morocco	*cytb* , *12S*	DQ451984/DQ452001
Hemorrhois hippocrepis	DQ452002/DQ451964	Beni Arouss, Morocco	*cytb* , *12S*	DQ451964/DQ452002
Hemorrhois hippocrepis	DQ452003/DQ451975	Marrackech, Morocco	*cytb* , *12S*	DQ451975/DQ452003
Hemorrhois hippocrepis	DQ451994/DQ451971	Algiers, Algeria	*cytb* , *12S*	DQ451971/DQ451994
Hemorrhois hippocrepis	DQ451995/DQ451972	Algeria	*cytb* , *12S*	DQ451972/DQ451995
Hemorrhois hippocrepis	DQ451996/DQ451973	Tlemcen, Algeria	*cytb* , *12S*	DQ451973/DQ451996
Hemorrhois hippocrepis	DQ452005/DQ451987	Nebeur, Tunisia	*cytb* , *12S*	DQ451987/DQ452005
Hemorrhois hippocrepis	DQ452004/DQ451985	Nebeur, Tunisia	*cytb* , *12S*	DQ451985/DQ452004
Hemorrhois hippocrepis	DQ452006/DQ451986	Hammam Bourguiba, Tunisia	*cytb* , *12S*	DQ451986/DQ452006
Malpolon monspessulanus	DB11859	Acudia, Mallorca	*cytb*	KP036593
Malpolon monspessulanus	DQ451880	Greece	*cytb*	DQ451880
Malpolon monspessulanus	DQ451881	Greece	*cytb*	DQ451881
Malpolon monspessulanus	DQ451882	Greece	*cytb*	DQ451882
Malpolon monspessulanus	DQ451883	Greece	*cytb*	DQ451883
Malpolon monspessulanus	DQ451884	Vrysoulles, Cyprus	*cytb*	DQ451884
Malpolon monspessulanus	DQ451885	Tabarka, Tunisia	*cytb*	DQ451885
Malpolon monspessulanus	DQ451886	Tozeur city, Tunisia	*cytb*	DQ451886
Malpolon monspessulanus	DQ451887	El Cairo, Egypt	*cytb*	DQ451887
Malpolon monspessulanus	DQ451888	Tanger, Morocco	*cytb*	DQ451888
Malpolon monspessulanus	DQ451899	Kenitra, Morocco	*cytb*	DQ451899
Malpolon monspessulanus	DQ451902	Saidia, Morocco	*cytb*	DQ451902
Malpolon monspessulanus	DQ451903	Ras el Ma, Morocco	*cytb*	DQ451903
Malpolon monspessulanus	DQ451922	Mellila, Morocco	*cytb*	DQ451922
Malpolon monspessulanus	DQ451925	Essaouira, Morocco	*cytb*	DQ451925
Malpolon monspessulanus	AY643396	Morocco	*cytb*	AY643396
Malpolon monspessulanus	DQ451893	El Aouedj, Algeria	*cytb*	DQ451893
Malpolon monspessulanus	DQ451919	Chrea, Algeria	*cytb*	DQ451919
Malpolon monspessulanus	DQ451889	Barbate, Spain	*cytb*	DQ451889
Malpolon monspessulanus	DQ451890	Jerez de la Frontera, Spain	*cytb*	DQ451890
Malpolon monspessulanus	DQ451891	Sierra de Retin, Spain	*cytb*	DQ451891
Malpolon monspessulanus	DQ451892	Marismas del Guadalquivir, Spain	*cytb*	DQ451892
Malpolon monspessulanus	DQ451894	Bodegones, Spain	*cytb*	DQ451894
Malpolon monspessulanus	DQ451895	Cadiz, Spain	*cytb*	DQ451895
Malpolon monspessulanus	DQ451897	Cadiz, Spain	*cytb*	DQ451897
Malpolon monspessulanus	DQ451901	Cadiz, Spain	*cytb*	DQ451901
Malpolon monspessulanus	DQ451911	Cadiz, Spain	*cytb*	DQ451911
Malpolon monspessulanus	DQ451896	Ceuta, Spain	*cytb*	DQ451896
Malpolon monspessulanus	DQ451920	Ceuta, Spain	*cytb*	DQ451920
Malpolon monspessulanus	DQ451898	Sant Celoni, Spain	*cytb*	DQ451898
Malpolon monspessulanus	DQ451900	Badalona,Spain	*cytb*	DQ451900
Malpolon monspessulanus	DQ451904	Granada, Spain	*cytb*	DQ451904
Malpolon monspessulanus	DQ451912	Granada, Spain	*cytb*	DQ451912
Malpolon monspessulanus	DQ451916	Granada, Spain	*cytb*	DQ451916
Malpolon monspessulanus	DQ451905	Barrancos/Encinasola, Spain	*cytb*	DQ451905
Malpolon monspessulanus	DQ451906	Jaen, Spain	*cytb*	DQ451906
Malpolon monspessulanus	DQ451910	Jaen, Spain	*cytb*	DQ451910
Malpolon monspessulanus	DQ451907	Huelva, Spain	*cytb*	DQ451907
Malpolon monspessulanus	DQ451908	Almeria, Spain	*cytb*	DQ451908
Malpolon monspessulanus	DQ451909	Almeria, Spain	*cytb*	DQ451909
Malpolon monspessulanus	DQ451918	Almeria, Spain	*cytb*	DQ451918
Malpolon monspessulanus	DQ451923	Almeria, Spain	*cytb*	DQ451923
Malpolon monspessulanus	DQ451913	Huetor Santillan, Spain	*cytb*	DQ451913
Malpolon monspessulanus	DQ451924	Huetor Santillan, Spain	*cytb*	DQ451924
Malpolon monspessulanus	DQ451914	Sevilla, Spain	*cytb*	DQ451914
Malpolon monspessulanus	DQ451921	Sevilla, Spain	*cytb*	DQ451921
Malpolon monspessulanus	DQ451915	San Juan de Terreros, Spain	*cytb*	DQ451915
Malpolon monspessulanus	DQ451917	Padul, Spain	*cytb*	DQ451917
Macroprotodon mauritanicus	DB7364	Santa Eugenia, Mallorca	*cytb* , *12S*	KP036592, KP036563
Macroprotodon mauritanicus	DB7368	Palma, Mallorca	*cytb*	KP036591
Macroprotodon mauritanicus	DB11860	Mallorca	*cytb* , *12S*	KP036590, KP036562
Macroprotodon mauritanicus	DB11895	Mallorca	*cytb* , *12S*	KP036588, KP036561
Macroprotodon mauritanicus	DB13779	Menorca	*cytb*	KP036589
Macroprotodon mauritanicus	AY643359/AY643276	Mazagón, Spain	*cytb* , *12S*	AY643359/AY643276
Macroprotodon mauritanicus	AY643358/AY643275	Barbate, Spain	*cytb* , *12S*	AY643358/AY643275
Macroprotodon mauritanicus	AY643364/AY643281	Valle de Matamoros, Spain	*cytb* , *12S*	AY643364/AY643281
Macroprotodon mauritanicus	AY643363/AY643280	Huelva, Spain	*cytb* , *12S*	AY643363/AY643280
Macroprotodon mauritanicus	AY643362/AY643279	Benaocaz, Spain	*cytb* , *12S*	AY643362/AY643279
Macroprotodon mauritanicus	AY643361/AY643278	Puebla de Guzman, Spain	*cytb* , *12S*	AY643361/AY643278
Macroprotodon mauritanicus	AY643360/AY643277	Gandul, Spain	*cytb* , *12S*	AY643360/AY643277
Macroprotodon mauritanicus	AY643385/AY643302	Mallorca, Spain	*cytb* , *12S*	AY643385/AY643302
Macroprotodon mauritanicus	AY643384/AY643301	Mallorca, Spain	*cytb* , *12S*	AY643384/AY643301
Macroprotodon mauritanicus	AY643366/AY643283	Tetouan, Morocco	*cytb* , *12S*	AY643366/AY643283
Macroprotodon mauritanicus	AY643365/AY643281	Tetouan, Morocco	*cytb* , *12S*	AY643365/AY643281
Macroprotodon mauritanicus	AY643367/AY643284	Ashila, Morocco	*cytb* , *12S*	AY643367/AY643284
Macroprotodon mauritanicus	AY643369/AY643286	Fez, Morocco	*cytb* , *12S*	AY643369/AY643286
Macroprotodon mauritanicus	AY643368/AY643285	Fez, Morocco	*cytb* , *12S*	AY643368/AY643285
Macroprotodon mauritanicus	AY643370/AY643287	Cap des Trois Fourches, Morocco	*cytb* , *12S*	AY643370/AY643287
Macroprotodon mauritanicus	AY643371/AY643288	Amersid, Morocco	*cytb* , *12S*	AY643371/AY643288
Macroprotodon mauritanicus	AY643372/AY643289	Amersid, Morocco	*cytb* , *12S*	AY643372/AY643289
Macroprotodon mauritanicus	AY643373/AY643290	Amersid, Morocco	*cytb* , *12S*	AY643373/AY643290
Macroprotodon mauritanicus	AY643379/AY643296	Tizi-n-Tichka, Morocco	*cytb* , *12S*	AY643379/AY643296
Macroprotodon mauritanicus	AY643378/AY643295	Sidi Ifni, Morocco	*cytb* , *12S*	AY643378/AY643295
Macroprotodon mauritanicus	AY643375/AY643292	Ifrane, Morocco	*cytb* , *12S*	AY643375/AY643292
Macroprotodon mauritanicus	AY643374/AY643291	Naour, Morocco	*cytb* , *12S*	AY643374/AY643291
Macroprotodon mauritanicus	AY643376/AY643293	Azrou, Morocco	*cytb* , *12S*	AY643376/AY643293
Macroprotodon mauritanicus	AY643377/AY643294	Essaouira, Morocco	*cytb* , *12S*	AY643377/AY643294
Macroprotodon mauritanicus	AY643382/AY643299	Beni Snassen, Morocco	*cytb* , *12S*	AY643382/AY643299
Macroprotodon mauritanicus	AY643383/AY643300	Beni Snassen, Morocco	*cytb* , *12S*	AY643383/AY643300
Macroprotodon mauritanicus	AY643380/AY643297	Tarofalt, Morocco	*cytb* , *12S*	AY643380/AY643297
Macroprotodon mauritanicus	AY643381/AY643298	E. Molouya estuary, Morocco	*cytb* , *12S*	AY643381/AY643298
Macroprotodon mauritanicus	AY643390	El Agheila, Lybia	*cytb*	AY643390
Macroprotodon mauritanicus	AY643389/AY643306	Médjana, Algeria	*cytb* , *12S*	AY643389/AY643306
Macroprotodon mauritanicus	AY643388/AY643305	Ain Draham, Tunisia	*cytb* , *12S*	AY643388/AY643305
Macroprotodon mauritanicus	AY643387/AY643304	Bou Chebka, Tunisia	*cytb* , *12S*	AY643387/AY643304
Macroprotodon mauritanicus	AY643386/AY643303	Tabarka, Tunisia	*cytb* , *12S*	AY643386/AY643303
Rhinechis scalaris	20904	Alicante	*cytb*	KP036600
Rhinechis scalaris	EU497634	Barcelos, Braga	*cytb*	EU497634
Rhinechis scalaris	EU497634	Castelo Branco	*cytb*	EU497634
Rhinechis scalaris	EU497634	Resende, Viseu	*cytb*	EU497634
Rhinechis scalaris	EU497634	Sintra, Lisboa	*cytb*	EU497634
Rhinechis scalaris	EU497634	Motilla del Palancar, Cuenca	*cytb*	EU497634
Rhinechis scalaris	EU497634	Vila Nova de Paiva, Viseu	*cytb*	EU497634
Rhinechis scalaris	EU497634	Laroya, Almeria	*cytb*	EU497634
Rhinechis scalaris	EU497634	Ribeira da Pena, Vila Real	*cytb*	EU497634
Rhinechis scalaris	EU497634	Fermoselle, Zamora	*cytb*	EU497634
Rhinechis scalaris	EU497634	Vimioso, Bragança	*cytb*	EU497634
Rhinechis scalaris	EU497634	Fornillos, Zamora	*cytb*	EU497634
Rhinechis scalaris	EU497634	Monte La Algaida, Seville	*cytb*	EU497634
Rhinechis scalaris	1814	Peñas de San Pedro, Zamora	*cytb*	KP036597
Rhinechis scalaris	1834	El Carrascal, Avila	*cytb*	KP036599
Rhinechis scalaris	12002	Ibiza	*cytb*	KP036598
Rhinechis scalaris	9250	Sant Joan, Mallorca	*cytb*	KP036594
Rhinechis scalaris	7076	Menorca	*cytb*	KP036596
Rhinechis scalaris	7138	Menorca	*cytb*	KP036595
